# Isolating the Contributions of Resistance-Ratio Normalization and Humidity Fusion in a Minimal Three-Sensor Electronic Nose

**DOI:** 10.3390/s26206645

**Published:** 2026-10-21

**Authors:** Tharun H. Nambi, Nikhil Bhimireddy, Joseph P. McElroy

**Affiliations:** 1 Olentangy Orange High School, Lewis Center, OH 43035, USA; 2 Pelotonia Institute for Immuno-Oncology, The Ohio State University Wexner Medical Center, Columbus, OH 43210, USA; 3 Program in Liberal Medical Education, Brown University, Providence, RI 02912, USA; nikhil_bhimireddy@brown.edu; 4 Department of Biomedical Informatics, The Ohio State University, Columbus, OH 43210, USA; joseph.mcelroy@osumc.edu

**Keywords:** chemical sensors, electronic noses, embedded systems, gas sensors, humidity, machine learning, sensor arrays, sensor fusion, signal processing, volatile organic compounds

## Abstract

**Highlights:**

**What are the main findings?**

- On a single device, converting divider voltage to sensor resistance is a monotone transform that a random forest cannot see; the whole effect of R_s_/R_0_ normalization is the division by R_0_, which costs 3.8 points for a random forest and 8–9 points for linear classifiers, a cost to which incomplete baseline recovery may contribute.
- The dominant residual error, a glycerol–water confusion, is best explained as physical rather than as a signal-conditioning artifact; admitting a humidity channel resolves it, while temperature and eCO_2_ do not.

**What are the implications of the main findings?**

- On the array and panel tested here, where the residual error traced to a variable the array does not measure, adding a transducer for that variable was more effective than transforming channels that do not encode it.
- Design choices that the low-cost electronic nose literature applies together should be ablated individually; a deliberately minimal array is what makes each contribution measurable.

**Abstract:**

Low-cost metal oxide semiconductor (MOS) electronic noses often combine resistance-ratio (R_s_/R_0_) normalization with a humidity transducer, both from the outset, so neither is measured. We isolate each on a three-sensor MQ array across nine pure headspaces. On one device the voltage-to-resistance step is monotone, so on 245 trials with per-trial clean-air baselines, the whole effect is the R_0_ division: it cost a random forest 3.8 percentage points (seed-sweep interval 2.3–5.3) and linear models 8–9 points, possibly inflated by incomplete baseline recovery, and left the dominant error mode, a symmetric glycerol–water confusion, untouched. In-chamber humidity, from a channel withheld from the classifier, points to a physical rather than signal-conditioning origin. Admitting humidity as a fourth feature raises accuracy from 90.2 to 99.3% (450 trials, ten seeds, *p* < 10^−8^) and from 86.7 to 93.3% under a cross-day holdout; temperature and equivalent CO_2_ (eCO_2_) do not. A leave-one-sensor-out test shows MQ-9 adds 0.89 ± 0.63 points on three channels and nothing with humidity admitted, so two MOS channels and humidity suffice here. The contribution is the matched ablation, not the accuracy: we propose, for MOS arrays sampling aqueous preparations, adding a transducer for the interferent rather than transforming channels that cannot resolve it.

## 1. Introduction

Gas chromatography–mass spectrometry (GC–MS) resolves volatile organic compounds (VOCs) with molecular specificity and remains the reference technique for headspace analysis. Instrument cost, operator training, and sample preparation confine it to centralized laboratories, however, and preclude the distributed or continuous deployment required in industrial process control, occupational safety, and environmental hazard monitoring.

Electronic noses address a different problem. Rather than resolving individual molecules, arrays of cross-reactive gas sensors combined with pattern-recognition algorithms characterize the composite chemical signature of a headspace [1,2]. Metal oxide semiconductor (MOS) transducers, and the MQ series in particular, are widely used in this role because of low unit cost, long operational lifetime, and broad sensitivity to combustible and volatile species [3,4]. This combination has made low-cost electronic nose prototypes an active area of development across food quality, environmental, and biomedical contexts [5,6,7,8], including exploratory work on exhaled-breath diagnostics [9,10,11,12], although the present study is confined to controlled chemical headspaces and makes no clinical claim.

Two engineering problems recur in this literature and limit the reproducibility of reported results. First, raw analog output voltage is not, in general, a portable measurand: it is set by a voltage divider whose load resistance R_L_ varies between boards, and it drifts with ambient temperature, humidity, and heater aging [13,14,15]. Humidity interference is a distinct drift mechanism in which water vapor competes with target analytes for adsorption sites and shifts baseline resistance nonlinearly [13], and is treated as such in recent surveys of drift compensation for these arrays [16]. Converting to sensor resistance and normalizing against a clean-air baseline (R_s_/R_0_) is the standard mitigation in the MOS sensor literature, in which sensor response is conventionally expressed as a power law in R_s_/R_0_ [17,18]. Second, many prototypes delegate classification to cloud services, which introduces inference latency and a hard dependency on network connectivity that is impractical in remote or industrial settings.

### 1.1. Related Work and the Gap This Study Addresses

Published low-cost MOS electronic noses typically employ substantially larger arrays, commonly five to sixteen sensors, and report performance on datasets acquired for a single application domain with classification performed off-device [19,20,21,22,23,24,25]. Recent builds continue this pattern: War et al. pair a five-sensor MQ array including the MQ-3 and MQ-135 used here with separate humidity and temperature transducers and apply conductance-ratio normalization [26], the combination this work evaluates directly. Mousa et al. combine two MQ channels with temperature and humidity inputs to a compact neural network and report 97.3% across four gas classes, with voltage ratios among the engineered features [27]; there too, the two conditioning steps are applied together. Beyond the systems in Table 1, the wider literature treats both as established practice: resistance-ratio referencing follows from the power-law response of the sensing layer [17], while humidity compensation, from response-mechanism models to temperature modulation with discriminant analysis, remains an active current literature [28,29]. Array reduction has been studied directly; Zhang et al. removed channels from an eight-sensor MQ array by significance and found little loss down to four [30]. The nearest comparator in array size is the study by Singh et al., whose three-sensor metal oxide array resolves ethanol, acetone, toluene and chloroform in mixtures at better than 99% with a random forest [31], again with no ablation of either design choice. Humidity compensation, in which a model of the humidity response is used to correct the MOS signal, is the established remedy for water-vapor interference [13,16,32]. Table 1 summarizes representative systems.

Two patterns are visible in Table 1. Arrays are larger, typically five to sixteen channels against the three used here, and inference is performed away from the acquisition hardware. Class counts are not directly comparable, since the task differs by application domain, and are given only to characterize each study. Auxiliary temperature and humidity transducers are routine in these builds, two of the systems in Table 1 included, so admitting such a channel is not in itself novel, and we do not claim that it is. What is absent, from the systems in Table 1 and from the wider literature surveyed here, is a measurement of what either design choice contributes. Where a humidity channel is present, it is supplied to the classifier from the outset. Where normalization is applied, it is applied throughout, so its effect is never measured against an unconditioned baseline on the same trials.

This study takes the opposite approach on both counts. The array, designated SCENT here, is deliberately minimal: three MQ channels with all computation on-device, so that the effect of each addition is large enough to measure. Normalization is ablated against raw voltage on matched trials and folds. The humidity channel is first withheld from the classifier and used only as independent evidence about the error mechanism, then admitted against a matched baseline as a test of the prediction that evidence generates, and finally checked for specificity against the two other auxiliary channels on the deck.

### 1.2. Contributions and Scope

This paper reports three findings, and we state their scope at the outset so that the headline figures are read correctly.

- On this array, resistance-ratio normalization reduces to a division by the per-trial clean-air baseline, because the voltage-to-resistance step is monotone; that division imposed a consistent cost, small and seed-sensitive for a random forest and large and robust for linear classifiers, to which incomplete baseline recovery may contribute (Section 4.1), and left the dominant error mode unchanged.
- That error mode, a symmetric glycerol–water confusion, is better explained as physical than as an artifact of signal conditioning. Independent humidity measurements point to the mechanism before any humidity feature is used.
- Admitting the humidity channel as a fourth feature resolves the confusion, as the mechanism predicts, and two other auxiliary channels admitted in its place do not.

The nine-analyte panel was chosen to span chemical classes, and seven of the nine headspaces are resolved at 98–100% precision and recall by three MOS channels alone. The classification task is therefore not difficult, and we do not present the final accuracy as evidence of a capable instrument. The task’s value is that it leaves exactly one confusion, which makes it possible to attribute changes in accuracy to specific causes. The contribution of this work is that attribution. On-device inference is a property of the platform rather than a contribution we claim. It is reported in Section 4.4 because it constrains the design, and it is not one of the three findings above. Current work in that area is considerably more ambitious than ours, for instance, quantized networks designed for on-device drift correction without recalibration [33].

## 2. Materials and Methods

### 2.1. Sensing Array and Compute

The platform was constrained to USD 83 in total component cost. That figure is the component cost at the time of purchase, itemized in the repository. It excludes shipping, tax, tools and the 3D printer, and the ADC and the two digital sensors are generic breakouts rather than branded equivalents, which cost roughly three times as much. A Raspberry Pi 5 single-board computer (Raspberry Pi Ltd., Cambridge, UK) served as the compute module, selected for adequate edge-inference throughput at low power draw. The sensing array comprised three MQ-series MOS sensors, MQ-3, MQ-9, and MQ-135 (Zhengzhou Hanwei Electronics Co., Ltd., Zhengzhou, China), chosen for broad overlapping sensitivity to alcohols, combustible aliphatics and carbon monoxide, and ammonia, respectively. Sensor-response behavior and its dependence on operating conditions follow the mechanisms described for tin dioxide transducers [4,34]. Because the Raspberry Pi provides no native analog input, MQ sensor outputs were digitized by an ADS1115 16-bit analog-to-digital converter (ADC; Texas Instruments, Dallas, TX, USA; generic breakout board) communicating over I^2^C, read single-ended on inputs A0–A2 at a programmable-gain setting of 1 (±4.096 V full scale).

**Figure 1:**
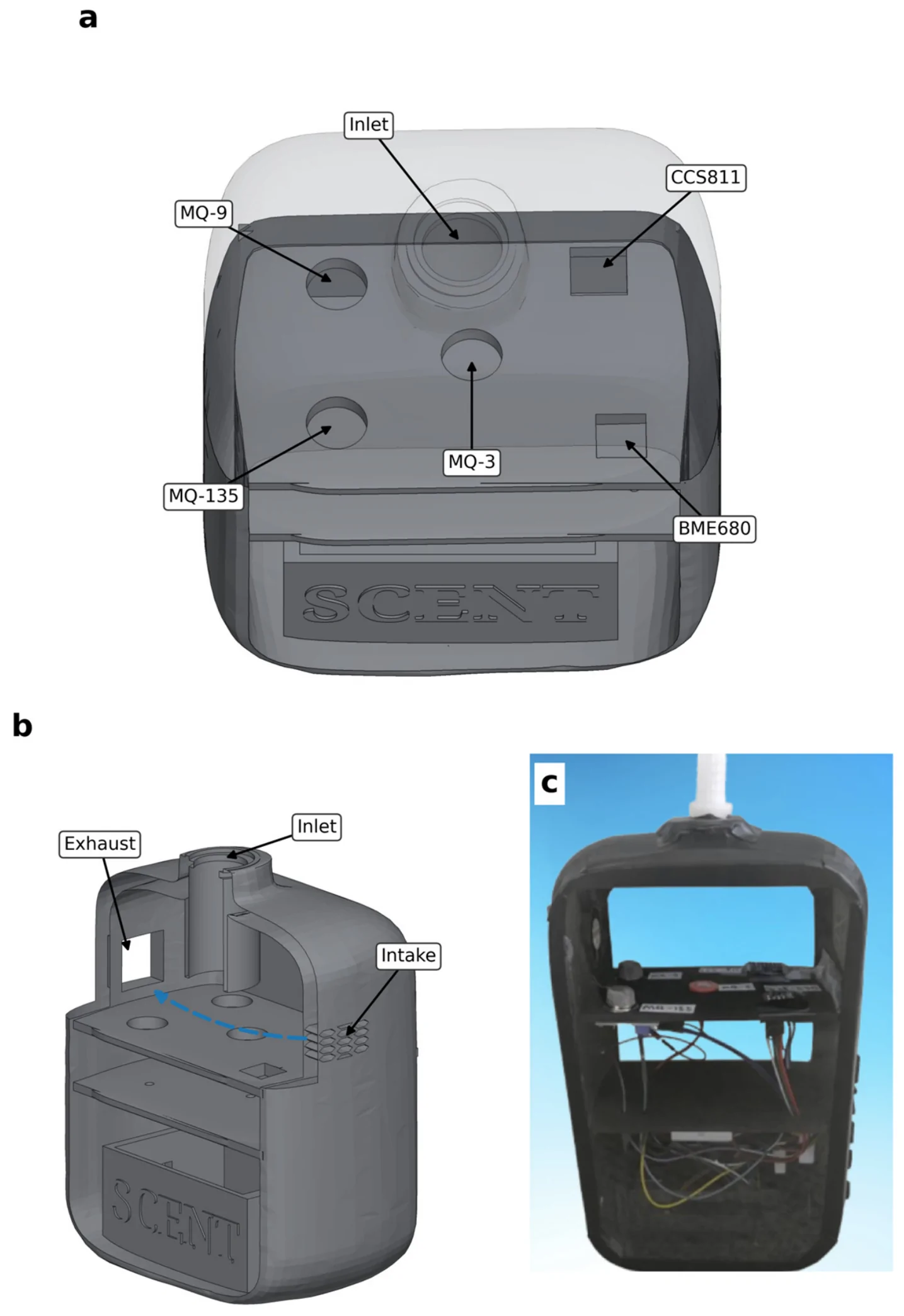
SCENT sampling chamber. (**a**) Sensor deck, rendered from the computer-aided design (CAD) model with the upper chamber shown semi-transparent: MQ-3, MQ-9, and MQ-135 supply the classifier, CCS811 and BME680 are logged but not classified, and sample vapor enters through the lid inlet. (**b**) Cutaway view with the front of the sampling chamber removed: during purge, an exhaust fan draws ambient air in through the honeycomb intake vent and out the opposing exhaust port (dashed arrow), establishing a cross-flow over the sensor deck. (**c**) Fabricated device printed in PETG, showing the populated sensor deck and the compute deck below.

Two additional digital sensors were mounted on the same deck and logged alongside the array: a BME680 (Bosch Sensortec, Reutlingen, Germany; generic breakout board) recording in-chamber temperature and relative humidity (RH), and a CCS811 (ams AG, Premstaetten, Austria; generic breakout board) recording equivalent carbon dioxide (eCO_2_) and total volatile organic compounds (TVOC). Neither was supplied to the classifier for the primary result. The CCS811 environmental-compensation register (ENV_DATA) was never written, so its TVOC and eCO_2_ outputs are independent of the BME680 readings. The CCS811 TVOC channel was withheld from all feature sets and reported results, for the reason given in Section 4.6. The eCO_2_ and temperature channels show ordinary overlapping distributions and are evaluated in Section 3.7. The BME680 humidity channel becomes central to the results reported below; its exclusion from the primary feature set is what makes it usable as independent evidence. Sensor positions are shown in Figure 1. Acquisition was performed by a Python script running on the Raspberry Pi, which drives all three phases of the trial protocol, samples every channel at 1 Hz, and writes one record per trial.

New sensors were conditioned by powering the heaters continuously for 48 h before any data collection, exceeding the manufacturer-specified preheat interval of over 24 h [35] and allowing the tin dioxide sensing layers to reach stable operating characteristics. The array was additionally warmed up for 30 min at the start of each acquisition session before the first trial.

### 2.2. Sampling Chamber and Purge Fluidics

**Figure 2:**
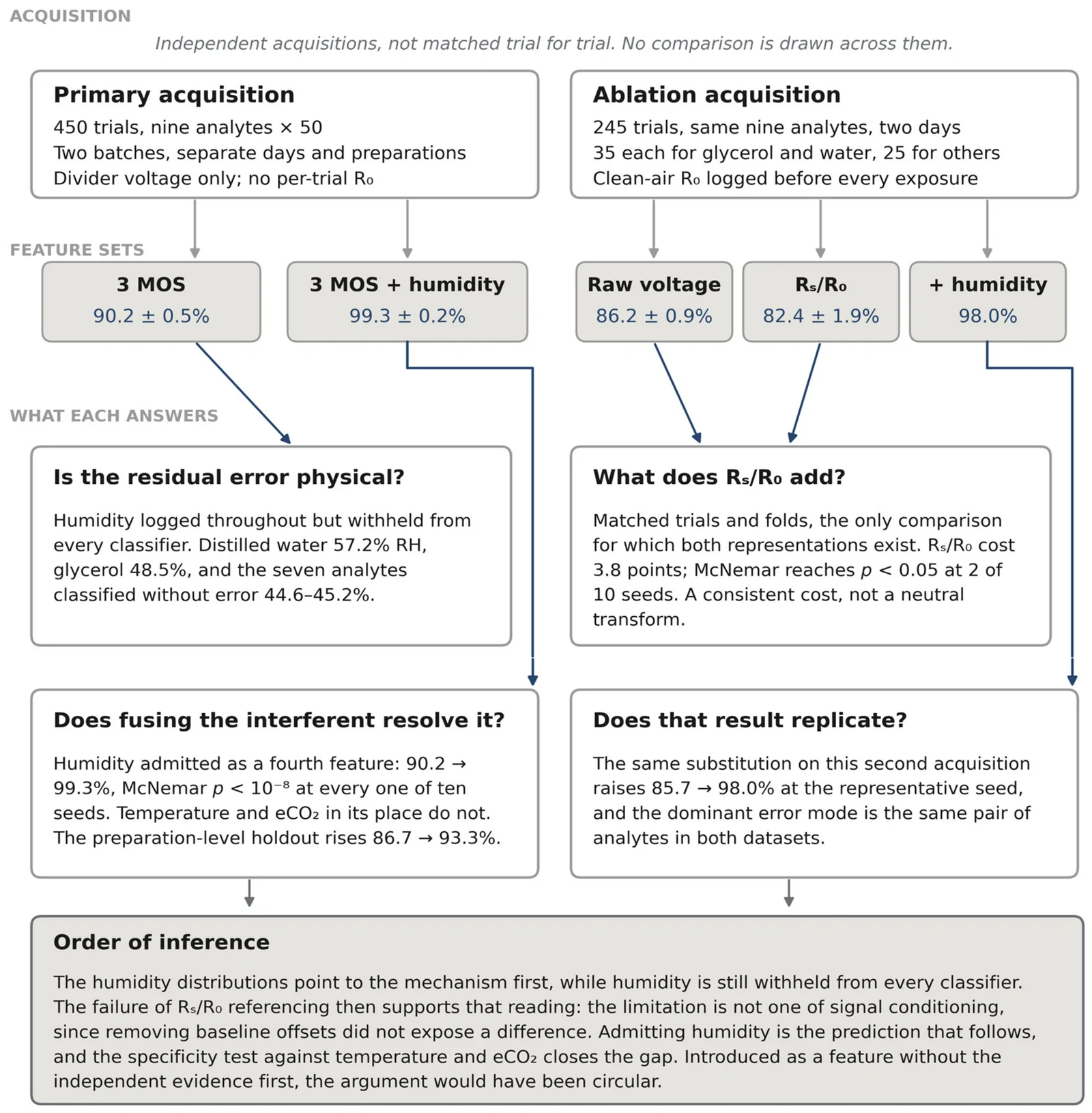
Analysis scheme. The two independent acquisitions, the feature sets derived from each, and the question each one answers. The primary acquisition carries no per-trial R_0_ baseline, so the normalization comparison is possible only on the ablation acquisition; the two are not matched trial for trial, and no comparison is drawn across them. The lower panel gives the order of inference: the humidity distributions point to the mechanism while humidity is still withheld from every classifier, which is what keeps the fusion result from being circular.

The sampling chamber was modeled in Autodesk Fusion 360 (Autodesk, Inc., San Francisco, CA, USA) and printed on an Elegoo Centauri Carbon 3D printer (ELEGOO, Shenzhen, China) in poly(ethylene terephthalate glycol) (PETG; generic filament purchased via AliExpress, Alibaba Group, Hangzhou, China). PETG was selected over poly(lactic acid) for chemical resistance and lower off-gassing; polymer off-gassing perturbs the clean-air baseline and is a known confound in 3D-printed sensing enclosures. Sample vapor enters through an inlet port in the chamber lid. During the recovery phase, an active exhaust fan (generic 2-pin fan purchased via AliExpress, Alibaba Group, Hangzhou, China) draws ambient air in through a honeycomb vent on one wall and out through an exhaust port on the opposite wall, establishing a cross-flow over the sensor deck that accelerates desorption from the heated sensing layers and restores baseline resistance. The opposed-wall geometry was chosen so that the flow path crosses the array rather than short-circuiting between adjacent apertures. Figure 2 summarizes the two acquisitions described in Section 2.3 and Section 2.4 and the analysis built on them.

### 2.3. Analyte Panel and Trial Protocol

Figure 2 sets out the analysis as a whole: which acquisition supports which claim, and in what order the inferences are made. Nine headspaces were evaluated: ethanol, acetone, ethyl acetate, acetic acid, ammonia, hydrogen peroxide, propylene glycol, glycerol, and distilled water as a humidity control. The panel was chosen to span alcohols, ketones, esters, organic acids, polyols, an inorganic base, and a reactive oxygen species, so that error structure could be read against chemical similarity. Because the classes are chemically dissimilar, most pairs are expected to be separable by a broad-sensitivity array; the panel is a diagnostic instrument for attributing error, not a benchmark of discriminative power (Section 1.2). All analytes were used as purchased, undiluted, without further preparation or solvent addition. All reagents were obtained from a single supplier (Alliance Chemical, Taylor, TX, USA) and used as received. The ammonia and hydrogen peroxide preparations are supplied as aqueous solutions; the remaining seven were used neat.

A fixed liquid volume of 5 mL was placed in a standardized receptacle and allowed to equilibrate for 30 min before sampling. Each trial comprised a 30 s baseline stabilization phase, a 60 s exposure phase, and a 120 s active purge recovery phase. Fifty trials were acquired per analyte, 450 in total. Trial order was randomized and interleaved across all nine analytes rather than blocked by analyte, so that any within-session drift in sensor baseline would be distributed across classes rather than confounded with class identity. Trials were acquired in two batches per analyte, comprising 40 and 10 trials, collected on separate days from separate sample preparations; this split supports the preparation-level cross-day holdout reported below. Trials were acquired in sessions of a few hours rather than continuously, each session preceded by the 30 min warm-up described in Section 2.1; the first batch spanned several such sessions. One volume and one equilibration time were used for every analyte, although the panel spans several orders of magnitude in vapor pressure, and the vapor-phase concentration reached at sampling was not independently verified for any analyte. Thirty minutes is unlikely to reach full equilibrium for the least volatile members of the panel, glycerol in particular, whose headspace carries the interpretation developed in Section 3.6 and Section 4.3. The protocol therefore fixes the preparation rather than the delivered concentration, and the results should be read accordingly.

In-chamber temperature and relative humidity were logged for every trial by the on-board BME680 and spanned 23.9–27.1 °C and 41.4–59.6% relative humidity across the full dataset, with means of 25.5 °C and 46.8% RH. These are uncontrolled laboratory conditions rather than a conditioned environment. That matters in both directions: it is representative of the deployment setting a low-cost array is intended for, and it means in-chamber humidity varies across trials, which is precisely the variation the normalization step is supposed to suppress.

Ammonia solution and hydrogen peroxide are corrosive and were handled in a ventilated area with appropriate personal protective equipment. Ethanol, acetone, and ethyl acetate are flammable and were kept away from the continuously powered sensor heaters except during controlled exposure.

### 2.4. Normalization Ablation Dataset

A second, independent acquisition was performed under a modified protocol in which the clean-air baseline resistance R_0_ of each channel was recorded immediately before every exposure, permitting resistance-ratio features to be computed on a per-trial basis. This dataset comprises 245 trials across the same nine analytes, acquired over two days: 35 trials each for glycerol and distilled water, the pair responsible for the dominant error mode in the primary dataset, and 25 trials for each of the remaining seven analytes. All other protocol parameters were unchanged. The unequal allocation concentrates statistical power on the comparison the ablation was designed to test. R_0_ was recorded at the end of the 30 s baseline phase of each trial, 150 s after the preceding exposure ended (the 120 s purge followed by the 30 s baseline). Recovery was not measured: the figure of up to 120 s given in Section 4.6 is a judgement from the live trace, which for strongly adsorbing analytes was sometimes still drifting when R_0_ was read, so for those analytes the logged baseline may not represent a fully recovered sensor. This bears directly on the normalization result and is treated as a confound in Section 4.1.

### 2.5. Feature Definitions

Sensor output was digitized as divider voltage via the ADS1115 converter. Divider voltage may be converted to sensor resistance according to (1):

(1) $$R_{s}=R_{L}\left[\frac{V_{c}}{V_{\mathrm{out}}}-1\right]$$

where V_c_ is the circuit supply voltage (5 V), and R_L_ is the board load resistance. Resistance-ratio features are formed by dividing R_s_ by the clean-air baseline resistance R_0_ of the same channel. Because R_0_ is logged in load-resistor units, R_L_ cancels in the ratio, and the feature is dimensionless; the manufacturer datasheet specifies sensor response in these ratio units [35].

Every channel was sampled at 1 Hz throughout each trial, and the value logged for a channel is the arithmetic mean of its samples over the 60 s exposure phase, 60 samples per trial. This applies uniformly to the three MQ divider voltages, to the BME680 relative-humidity and temperature readings, to the CCS811 eCO_2_ channel evaluated in Section 3.7, and to the values plotted in Section 3.7 and Section 3.8. Because the CCS811 returns eCO_2_ as an integer, averaging over the exposure phase is why the logged values for that channel are not integer-valued. One scalar per channel therefore represents each trial, and the shape of the response transient is discarded. Conclusions drawn below about what the array can and cannot separate apply to this representation rather than to the array in general; a representation retaining the transient may carry information this one does not. Current surveys of feature extraction for semiconductor gas sensors set out the alternatives [36], and a recent MOS electronic nose using 24 features per sensor across three operating temperatures illustrates what a richer representation costs in complexity [37].

The primary 450-trial dataset was classified on raw divider voltage, because a matched per-trial R_0_ baseline was not recorded alongside it. The headline accuracy must therefore not be read as an R_s_/R_0_ result. The raw-versus-normalized comparison is made on the 245-trial ablation dataset, where both representations are available for the same trials.

### 2.6. Classification and Validation

All model fitting and the cross-validated analyses reported here were performed offline in Python 3.12.3 using scikit-learn 1.8.0 [38]; the deployed instrument runs the same preprocessing and the fitted forest on the Raspberry Pi 5 itself, and the inference latency reported in Section 3.1 is measured on that device. Features were supplied to a random forest classifier [39] with 100 trees and all other hyperparameters at library defaults. Headline accuracies for the four principal feature sets (three MOS channels with and without humidity on the primary dataset; raw and R_s_/R_0_ features on the ablation dataset) are reported as mean ± s.d. across ten random seeds (0–9), each seed re-randomizing both the cross-validation fold assignment and the forest bootstrap, with folds aligned across feature sets within a seed so that paired tests remain valid; the per-seed range is given in Section 3.10. Every stochastic step uses a fixed random seed: seed 42 for the single-seed analyses and seeds 0–9 for the sweep, which is performed to quantify sensitivity to the partition of trials into folds and to the forest bootstrap. Where a fold-to-fold s.d. within a single seed is quoted, it is identified as such. Dispersion across the five cross-validation folds within a seed is reported as the population standard deviation, and dispersion across the ten seeds as the sample standard deviation. Confusion matrices, per-class precision and recall, per-fold accuracies, the classifier-family comparison, and the auxiliary-channel comparison are reported from a single representative seed (42) and are so labeled; the seed sweep shows that the primary-dataset figures move by less than two points across seeds, so these single-seed tables are representative. Each trial contributed a single feature row. No data were transformed, excluded, or treated as outliers; there were no missing values; and the datasets contain no duplicate feature rows.

Two validation schemes are reported throughout, and they estimate different things. Five-fold stratified cross-validation with shuffling, stratified by analyte class, treats the trial as the replication unit. Because trials within an acquisition batch derive from a shared sample preparation, this scheme can be optimistic, and its results should be read as upper bounds. A cross-day holdout, training on the first batch and testing on the second, treats the preparation as the replication unit; its results are conservative and are the figures we regard as the primary performance estimate. Reported cross-validation accuracy and macro-averaged F1 are the mean and standard deviation across the five folds. Per-class precision and recall and all confusion matrices are computed on pooled out-of-fold predictions, so every trial contributes exactly once as a test case, and no trial is scored by a model that saw it in training.

Two tests assessed performance against chance. An exact one-sided binomial test compared the number of correctly classified trials against the number expected under uniform random assignment across nine balanced classes. A label-permutation test with 1000 permutations, using identical folds and classifier settings, provided a distribution-free comparison; with 1000 permutations, the smallest attainable *p* value is 0.001. Paired comparisons between feature representations used McNemar’s test on out-of-fold predictions from the same trials, in its exact form at the representative seed and in its continuity-corrected chi-square form across the ten-seed sweep, with a paired *t*-test across fold accuracies as a secondary check. McNemar’s test assumes independent paired observations, and pooled out-of-fold predictions are not strictly independent, since they come from five models trained on overlapping data; its *p* values should therefore be read as approximate. For this reason, the normalization comparison is also summarized as the mean paired difference across the ten seeds with a 95% confidence interval (CI). Because the seeds re-randomize fold assignment on a single dataset, that interval describes the stability of the difference under resampling of folds rather than sampling variability across independent datasets. The significance threshold was 0.05 throughout.

Learning curves (Section 3.9) were computed by drawing a random subset of the training portion of each fold at the stated size, stratified by analyte class, fitting the classifier to that subset, and scoring the full held-out fold. Eight independent subset draws were averaged at each size within each fold, and the mean and standard deviation across the five folds are plotted. Fold assignment was held fixed across all training sizes so that the curve reflects training-set size alone.

**Figure 3:**
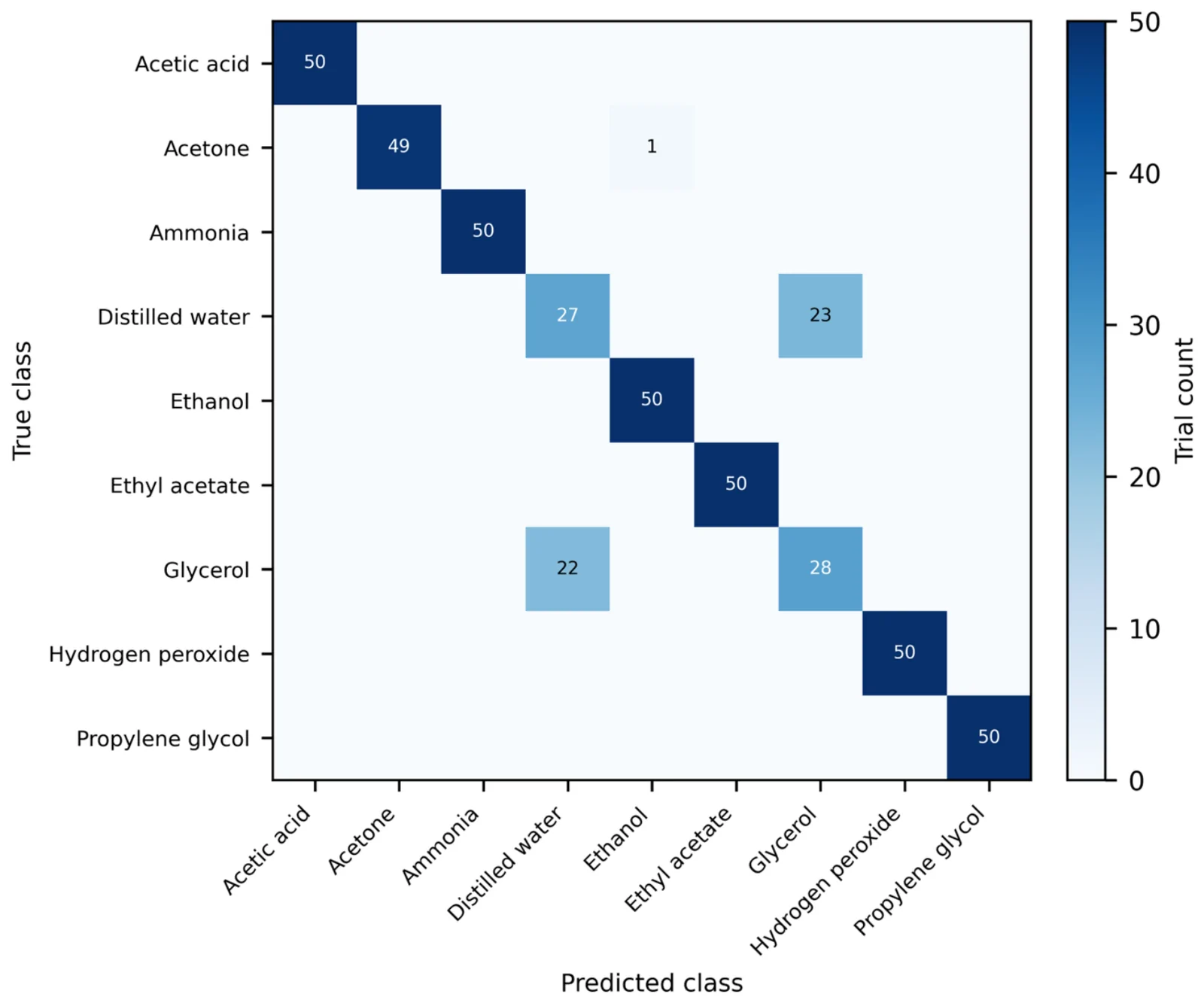
Confusion matrix for the nine-class headspace classification task on the three MOS channels, pooled out-of-fold predictions from five-fold stratified cross-validation (*n* = 450). Rows denote true class and columns denote predicted class; cell values are trial counts.

**Figure 4:**
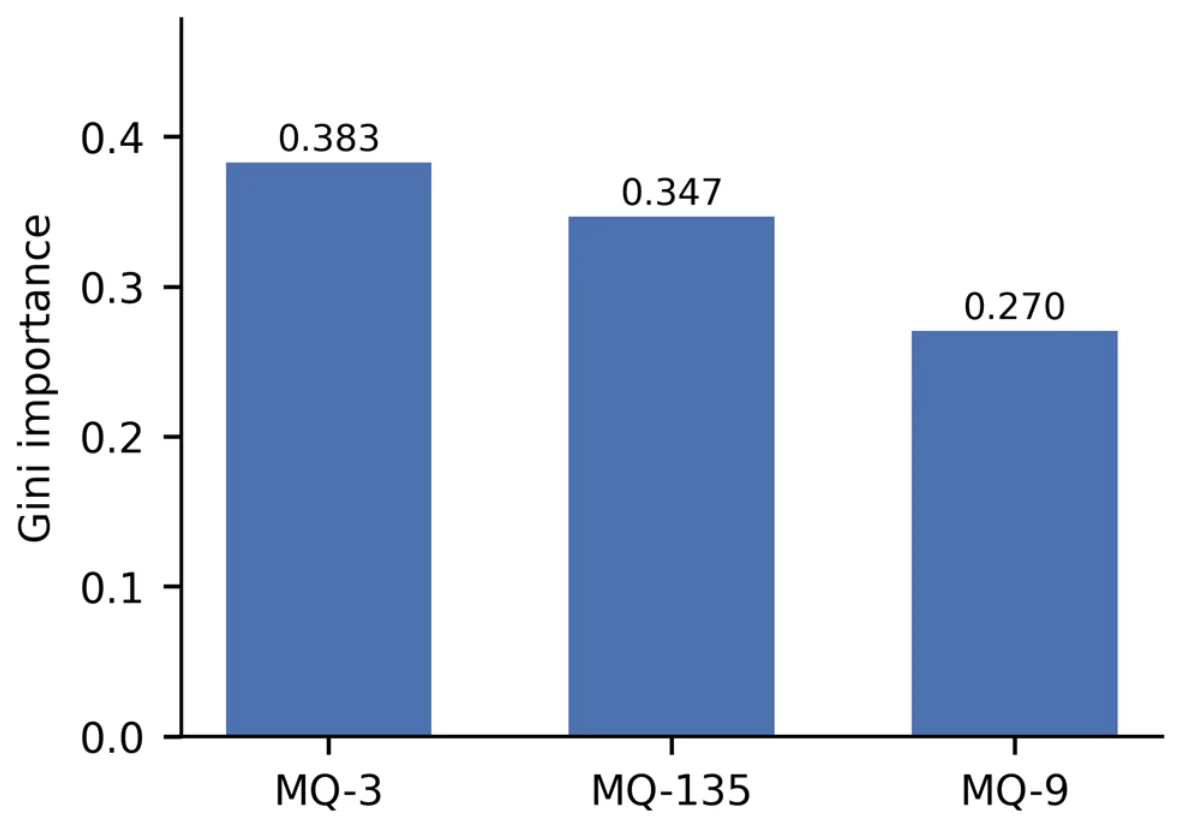
Gini impurity-based feature importance by sensor channel; random forest fitted on all 450 trials of the primary dataset.

**Figure 5:**
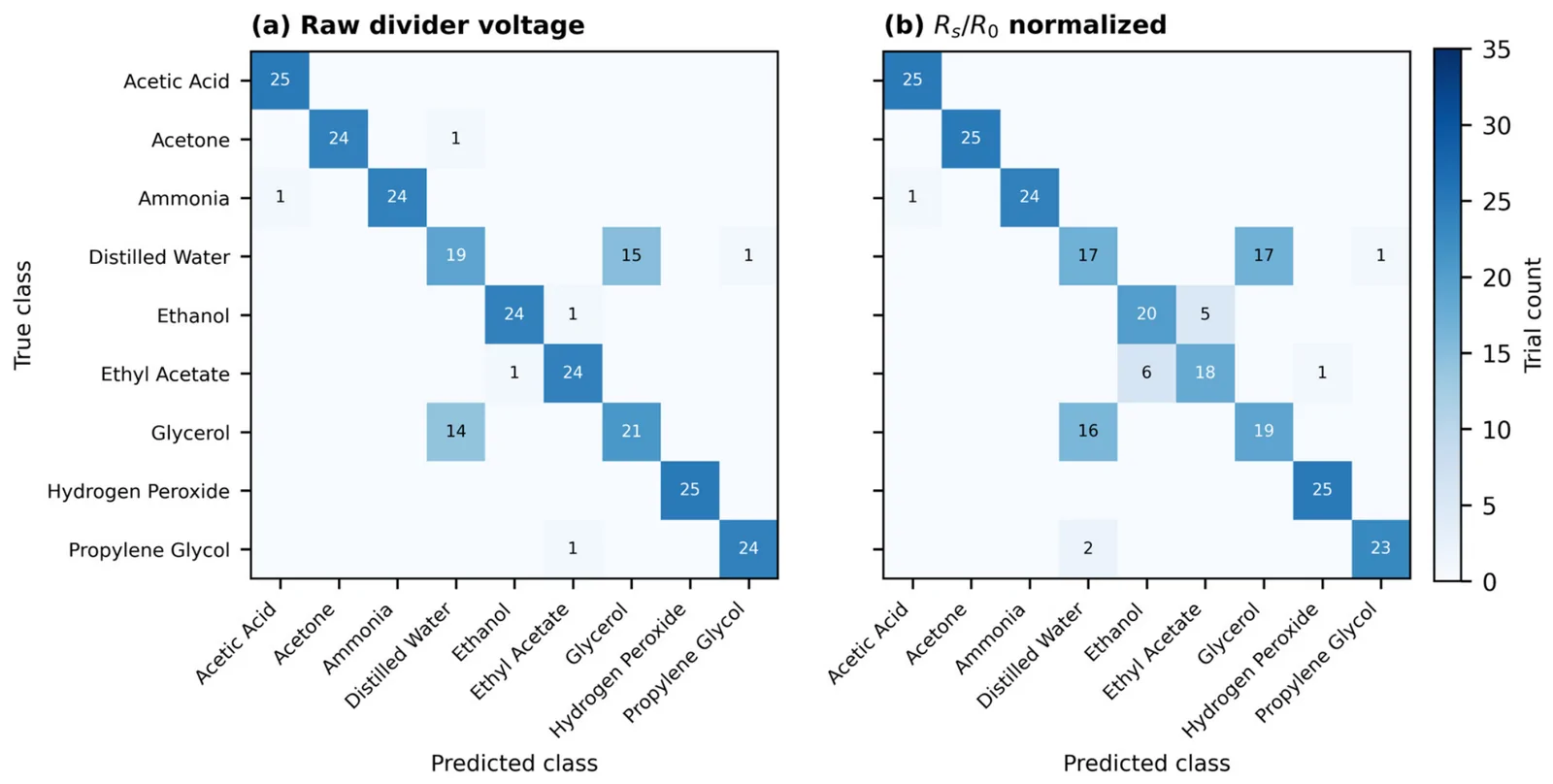
Confusion matrices for the normalization ablation; pooled out-of-fold predictions from five-fold stratified cross-validation (*n* = 245). (**a**) Raw divider voltage. (**b**) R_s_/R_0_ normalized features. The glycerol–water confusion persists under normalization, and an ethanol–ethyl acetate confusion that is marginal in (**a**), one trial each way, grows to five and six trials in (**b**).

**Figure 6:**
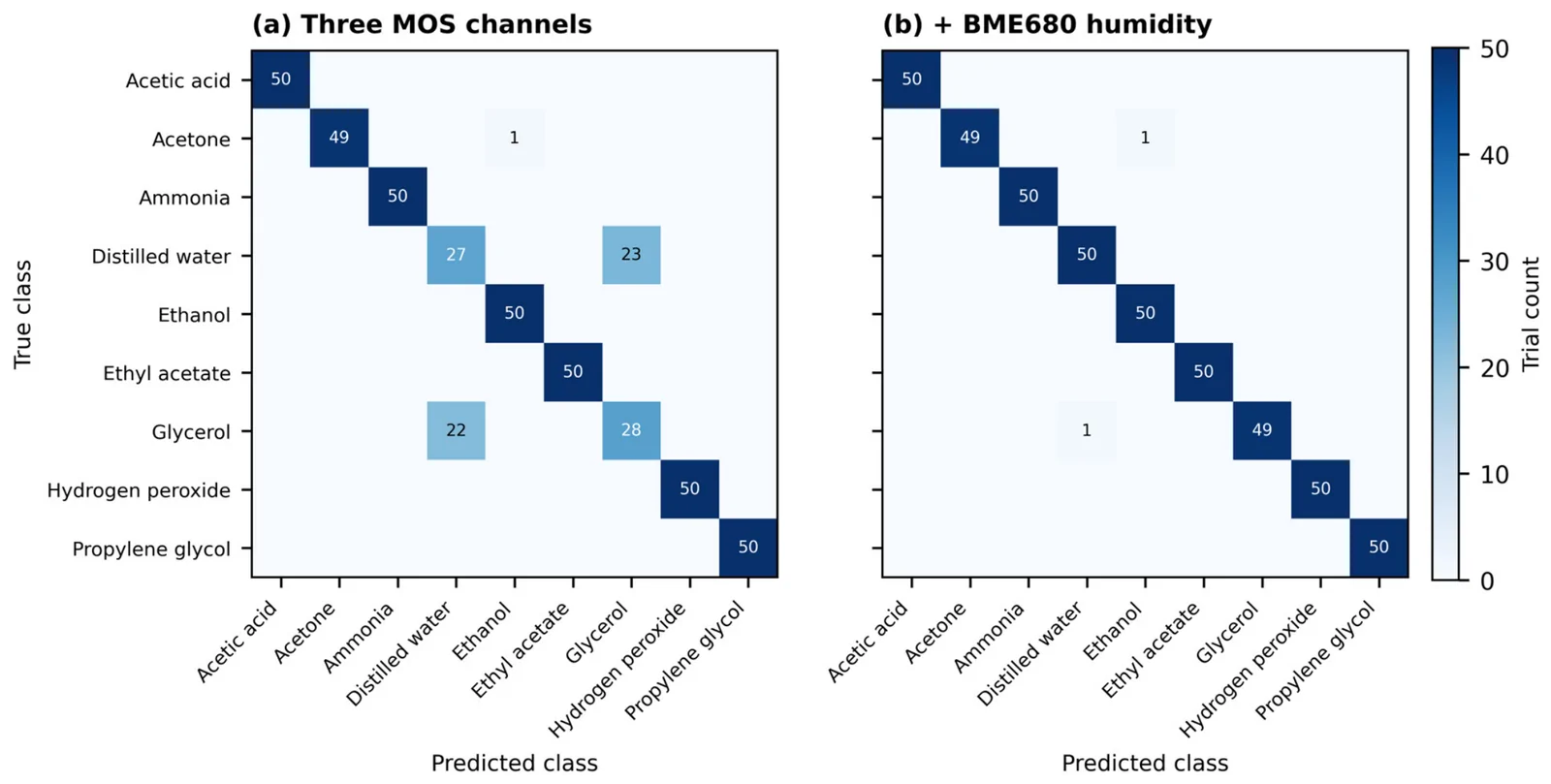
Effect of admitting the BME680 humidity channel as a fourth feature on the primary dataset (*n* = 450) using pooled out-of-fold predictions. (**a**) Three MOS channels. (**b**) Three MOS channels with humidity. The glycerol–water exchange collapses from 45 misassigned trials to one; two errors remain in the whole matrix.

To test whether the ensemble classifier was necessary, six alternative classifiers were fitted to the same features under identical folds, with feature standardization applied within each fold for the distance- and margin-based methods. Because a tree ensemble is invariant to monotone transformations of its features, the normalization ablation was also repeated with two scale-sensitive classifiers, logistic regression and a linear support vector machine, each with features standardized within each fold, and with a third representation, sensor resistance without the division by R_0_, to separate the two steps of the transform. The repository given in the Data Availability Statement contains the analysis script for the single-seed results, covering the three-channel and humidity-fusion classifications, the classifier comparison and the normalization ablation at the representative seed, and Figures 3–6; it also contains the four scripts behind the seed sweep, the leave-one-sensor-out ablation and its preparation-level repeat, the learning curve, and the CCS811 exclusion checks, each writing the values reported here.

### 2.7. Use of Generative Artificial Intelligence

A generative AI assistant (Claude Opus 5, released 24 July 2026, and, following its release on 22 September 2026, Claude Opus 5.5; Anthropic, San Francisco, CA, USA) was used, both in preparing the original submission and in preparing this revision, to write and run analysis code, to render figures from computed results, to assist literature retrieval, and to draft and edit manuscript text. All experimental design, hardware fabrication and data collection were performed by the authors, who also directed each analysis and interpreted the results. The analysis script for the single-seed results and the four scripts behind the seed sweep, the leave-one-sensor-out ablation, the learning curve and the CCS811 exclusion checks are all deposited in the public repository. The authors checked every reported value against the output of the analysis code and every citation against its primary source, and take full responsibility for the content of this article.

## 3. Results

### 3.1. Classification Performance, Three MOS Channels

The embedded random forest, trained on the three raw MQ-sensor divider voltages, achieved an overall accuracy of 90.2 ± 0.5% (range 89.3–90.9% across ten seeds) under five-fold stratified cross-validation across all 450 trials; at the representative seed, the accuracy was 89.8% with a fold-to-fold s.d. of 0.8 and a macro-averaged F1 of 0.897 ± 0.009. Chance performance for nine balanced classes is 11.1%. At the representative seed, 404 of 450 trials were correctly assigned against 50 expected under random assignment (exact binomial test, *p* < 0.001). The label-permutation test gave a permuted accuracy of 10.9 ± 1.8%, with a maximum permuted accuracy across 1000 permutations of 16.9%, far below the observed value; no permutation reached it (*p* = 0.001, the minimum attainable at this number of permutations).

The preparation-level estimate, training on the first acquisition batch (360 trials) and testing on the held-out second batch (90 trials) collected on a different day from a separate preparation, gave 86.7% accuracy. The two batches are independent acquisitions with separate sensor warm-up cycles and separate preparations. This is a single train-test comparison between the only two preparations available per analyte, so it carries no interval over preparations and does not establish robustness in general; it shows that the drop on one held-out preparation was 3.5 points. Inference latency, measured from the end of the exposure phase to class assignment, was under 1 s per trial on the Raspberry Pi 5. Per-class precision and recall are given in Table 2.

### 3.2. Structure of Classification Errors

Errors were structured rather than diffuse (Figure 3). The dominant error mode was a two-way confusion between glycerol and the distilled-water humidity control: 23 of 50 water trials were assigned to glycerol, and 22 of 50 glycerol trials were assigned to water, an essentially symmetric exchange. A single acetone trial was assigned to ethanol; no other misclassification occurred anywhere in the nine-by-nine matrix. No confusion arose between analytes drawn from chemically dissimilar groups, for example between ammonia and any oxygenated VOC. Seven of the nine classes were resolved at 98–100% precision and recall.

This error structure is what makes the rest of the analysis possible. With one confusion accounting for 45 of 46 errors, any intervention that changes accuracy must do so by acting on that pair, and its effect can be read directly from the two affected cells of the confusion matrix rather than inferred from an aggregate.

### 3.3. Sensor Channel Contribution

Gini impurity rankings extracted from the trained forest distribute the discriminative weight across all three channels, with MQ-3 carrying 38.3%, MQ-135 34.7%, and MQ-9 27.0% (Figure 4). Impurity importance measures how often a channel is selected for a split and how much it reduces impurity when it is; it does not measure whether the channel is necessary. Where two channels carry correlated information, the measure divides the credit between them, and each appears to contribute although either alone would suffice. Necessity can only be tested by refitting without the channel.

We therefore refit the classifier on every two-channel subset over the same ten seeds used elsewhere in this work (Supplementary Materials, Table S5). Removing MQ-3 cost 8.58 points and removing MQ-135 cost 2.62 points, both far outside the across-seed spread. Removing MQ-9 cost 0.89 ± 0.63 points, taken as the paired per-seed difference: a small cost, roughly a tenth of that for MQ-3, and on one of the ten seeds, the two-channel model was the more accurate. On the four-feature model that includes humidity, removing MQ-9 cost −0.13 ± 0.16 points, a negative cost meaning accuracy rose rather than fell, from 99.33 ± 0.18% to 99.47 ± 0.16%; the cost was never positive at any seed. Because shuffled cross-validation mixes trials from both preparations, it cannot establish that a channel is redundant on a preparation the model has not seen, so we repeated the ablation under the preparation-level holdout, training on the first batch and testing on the held-out second. Across ten forest seeds, the three-channel reference reached 87.0 ± 2.0% and the four-feature reference 94.3 ± 1.7%, intervals that contain the single-seed figures of 86.7% and 93.3% quoted elsewhere; the ± here reflects the forest seed on one train–test split, not variation across preparations. Removing MQ-9 cost −0.89 ± 2.61 points on three channels and −1.22 ± 1.92 on four, the reduced model being at least as accurate at eight of ten seeds in both cases, while removing MQ-3 cost 10.89 ± 2.39 and 8.22 ± 2.17. The direction agrees with the cross-validated result, but the held-out preparation comprises 90 trials and the intervals are correspondingly wide, so this test constrains the direction of the MQ-9 finding rather than its magnitude (Table S5c). Per-class F1 shows the same pattern (Table S5b). Dropping MQ-3 collapses acetone from 0.99 to 0.60 and propylene glycol from 1.00 to 0.65; dropping MQ-135 degrades six classes modestly; dropping MQ-9 leaves every class within 0.03 of its three-channel value.

MQ-9 is therefore close to redundant on three channels, and makes no measurable contribution once humidity is admitted, on this panel. Fitted alone, it reaches 35.2 ± 0.9%, against 77.0 ± 1.4% for MQ-3 and 65.4 ± 1.1% for MQ-135, so it carries the least independent information of the three and what it carries is largely recoverable from the other two. This does not contradict the cross-reactive array principle so much as locate its limit. Three MQ-series sensors with overlapping sensitivities to alcohols and combustible species do not supply three independent views of these nine headspaces. Zhang et al. report the same behavior on a larger MQ array, reducing eight channels to four at little cost [30].

The finding strengthens the case for a small array rather than weakening it. Two MOS channels and a humidity transducer reach 99.47 ± 0.16% under cross-validation and 95.6 ± 0.9% under the preparation-level holdout, in both cases above the corresponding figure for all four channels, at lower component cost. We retain the three-channel configuration in the reported results because it is the array that was built and characterized, and because the redundancy was established only by this ablation; an instrument designed from this result would carry MQ-3, MQ-135 and the BME680.

### 3.4. Comparison with Simpler Classifiers

Six alternative classifiers fitted to the same three features under identical cross-validation folds all performed comparably to the random forest, and several scored marginally higher (Table 3). Both support vector machine variants reached 91.8 ± 2.6% and a single unpruned decision tree reached 90.4 ± 1.1%, against 89.8 ± 0.8% for the forest. The spread across all seven models lies within approximately one standard deviation of the fold-to-fold variation, so these differences are not interpreted as meaningful separations between methods. The two support vector machine variants returned identical accuracy to four decimal places; their out-of-fold predictions differ on two trials, so this reflects coincident scores rather than a duplicated estimator. We did not pursue the preprocessing and resampling strategies reported to raise classification accuracy for sensor arrays, such as filtering with dimensionality reduction combined with synthetic oversampling [40]; on this panel, the classifier is not the binding constraint.

The relevant conclusion is that the ensemble buys nothing here. With three features and nine classes whose headspaces are largely separable, the reported accuracy reflects the information content of the sensor representation rather than the sophistication of the classifier, and the residual error is consistent with physical overlap between two headspaces rather than with decision-boundary complexity. The random forest is retained as the deployed model because its threshold splits are invariant to any monotonic rescaling of a feature, so no scaling step is required at inference time, and because it yields the impurity-based channel importances above; we note that this invariance concerns the feature scale and confers no robustness to baseline drift between training and deployment, which shifts values relative to fixed split thresholds; a linear model would be an equally defensible and computationally cheaper choice for embedded deployment. This also establishes the frame for the two results that follow: because model choice does not move the result, a change in accuracy under a different feature representation is attributable to the representation.

### 3.5. Effect of Resistance-Ratio Normalization

On the 245-trial ablation dataset, R_s_/R_0_ features gave 82.4 ± 1.9% accuracy (range 79.2–84.9% across ten seeds) against 86.2 ± 0.9% (85.3–87.3%) for raw divider voltage on identical trials and folds (Table 4). Paired by seed, raw voltage exceeded R_s_/R_0_ by 3.8 percentage points (95% CI 2.3–5.3); as set out in Section 2.6, this interval describes stability under resampling of folds, not sampling variability across datasets. At the trial level, the random-forest difference is less secure: McNemar’s test on paired out-of-fold predictions gave *p* between 0.03 and 1.00 across the ten seeds (median 0.24), reaching significance at two. At the representative seed, raw features were correct on 34 trials where normalized features failed, and normalized features were correct on 20 where raw features failed, with 176 correct under both and 15 wrong under both (*p* = 0.076). Two scale-sensitive classifiers show the same effect more strongly and more reliably. With features standardized within each fold, logistic regression lost 8.1 points under R_s_/R_0_ (95% CI 6.8–9.4; McNemar *p* < 0.05 at eight of ten seeds), and a linear support vector machine lost 9.0 points (95% CI 8.2–9.9; *p* < 0.05 at all ten). Converting voltage to resistance without dividing by R_0_ left the random forest unchanged to within four of 245 trials (1.6 points) at every seed and slightly improved both linear classifiers, by 1.6 and 2.0 points. The cost is therefore carried entirely by the division by R_0_. Why the random forest shows the smallest and least stable effect, and a confound that limits the comparison, are discussed in Section 4.1.

Normalization also left the dominant error untouched. At the representative seed, 16 of 35 glycerol trials and 17 of 35 water trials remained cross-assigned, and per-class F1 for the pair fell rather than rose, from 0.592 to 0.535 for glycerol and 0.551 to 0.486 for water. Mean recall across the seven analytes that had been classified essentially without error fell from 0.971 to 0.914. The degradation was not diffuse: normalization sharply amplified an ethanol–ethyl acetate confusion that is marginal under raw voltage, from one trial each way to five and six trials exchanged (Figure 5). The largest per-class losses were ethanol, 0.960 to 0.784, and ethyl acetate, 0.941 to 0.750. The preparation-level cross-day holdout, training on the 122 first-day trials and testing on the 123 second-day trials, preserved the ordering: 85.4% for raw features against 77.2% for normalized.

### 3.6. Humidity as a Fourth Feature

Glycerol boils at 290 °C [41], and its vapor pressure at ambient temperature is negligible, so the headspace above a glycerol sample is expected to contain very little glycerol vapor and to be dominated by water. We did not measure the vapor-phase composition, so this remains an inference from physical properties rather than a direct observation. The relevant variable is therefore the water associated with the preparation and with the chamber, not glycerol itself. The BME680 humidity channel, which is not among the features supplied to the classifier, supports this account directly. Mean relative humidity during acquisition was 57.2% for the distilled-water control and 44.6–45.2% for the seven analytes classified essentially without error, while glycerol trials fell between the two at 48.5%, with the single highest glycerol trial reaching into the water range (Section 3.8).

The two aqueous preparations warrant comment. Aqueous ammonia and hydrogen peroxide read 45.1% and 45.2% RH, indistinguishable from the neat organics and twelve points below the distilled-water control, despite both being predominantly water. Two accounts are consistent with this observation: the 60 s exposure may not reach headspace equilibrium for these preparations, or the dissolved solute may depress water activity enough to lower the equilibrium vapor pressure below that of the distilled-water control. The present data do not distinguish between them. We note also that capacitive polymer humidity elements are subject to cross-sensitivity from solvent vapors. The humidity-alone classification bounds how large that effect can be here: F1 lies between 0.077 and 0.182 across the seven analytes other than glycerol and water, so the channel carries almost no analyte-specific information beyond the pair it resolves.

That the confusion survives R_s_/R_0_ referencing is the central evidence for a physical rather than a signal-conditioning origin. Had the two headspaces differed in composition with that difference masked by baseline offsets, removing the offsets should have exposed it, and it did not, which places the limitation in what the three MOS channels measure rather than in how their signals are conditioned. The humidity measurements and the failure of normalization are consistent with this account, and we know of none that fits them better, but the vapor-phase composition was never characterized, so the account is supported rather than established.

This diagnosis makes a testable prediction, and it holds. Admitting the BME680 relative-humidity reading as a fourth feature, with the classifier and folds otherwise unchanged, raised accuracy on the primary dataset from 90.2 ± 0.5% to 99.3 ± 0.2% across ten seeds (range 99.1–99.6%), with McNemar *p* below 10^−8^ at every seed; on average, 41.7 trials were corrected against 0.5 broken. At the representative seed, 448 of 450 trials were correct. Glycerol and distilled water rose from F1 0.554 and 0.545 to 0.990 and 0.990, and the 45-trial exchange collapsed to a single glycerol trial assigned to water, with no water trial assigned to glycerol; only two errors remain in the entire matrix: that glycerol trial and the acetone-to-ethanol misassignment already present under three channels (Figure 6, Table 5). The same substitution raised the ablation dataset from 85.7% to 98.0% at the representative seed (fold-to-fold s.d. 5.3 and 2.6), and the preparation-level cross-day holdout from 86.7% to 93.3% (84 of 90 held-out trials). The cross-day gain is smaller than the cross-validated gain, which is expected: the second-day preparation is a single draw from preparation-level variation, and the holdout test set contains only ten trials per class.

Impurity-based importances for the four-feature model are MQ-3 0.346, MQ-135 0.338, and MQ-9 0.195, and relative humidity is 0.121, so humidity carries the smallest share of any channel. Because impurity-based importance is biased toward continuous features and is weak evidence on its own, we tested the claim directly by fitting the same classifier on relative humidity alone. Across nine classes, it reached 29.1 ± 3.3% accuracy against an 11.1% chance baseline, and the per-class structure is the informative part: F1 was 0.980 for distilled water and 0.692 for glycerol, but between 0.077 and 0.182 for the other seven analytes, at or near chance. Humidity alone therefore separates exactly the pair the MOS array cannot, and carries almost no information about anything else. It is not displacing the MOS array; it supplies the single dimension the array cannot resolve.

### 3.7. Specificity of the Humidity Gain

The array carries two further auxiliary channels, and admitting either in place of humidity tests whether the gain reflects the interferent specifically or simply the addition of a fourth input. At the representative seed, adding the BME680 temperature reading gave 90.9 ± 2.3% accuracy and adding the CCS811 eCO_2_ reading gave 90.9 ± 2.7%, against 89.8 ± 0.8% for the three MOS channels alone (Table 6; ± is fold-to-fold s.d.). Neither difference approaches significance under McNemar’s exact test (*p* = 0.33 and *p* = 0.36, respectively), and neither materially improved the glycerol–water pair: glycerol F1 reached 0.592 with temperature and 0.615 with eCO_2_, against 0.554 for the three channels and 0.990 with humidity. Supplying all three auxiliary channels (temperature, eCO_2_, and humidity) together gave 99.6 ± 0.5% at the same seed, identical to the three MOS channels with humidity. The eCO_2_ channel is a weaker control than temperature. It occupies a narrow band immediately above the 400 ppm floor of the device (401–496 ppm across all trials, against a specified range of 400–29,206 ppm [42]), so its dynamic range is compressed; it is not clipped, since every trial reads above the floor and 434 of 450 values are distinct. The comparison therefore shows that a fourth channel with a compressed dynamic range does not reproduce the humidity gain, rather than testing an alternative equally informative about the confused pair.

**Figure 7:**
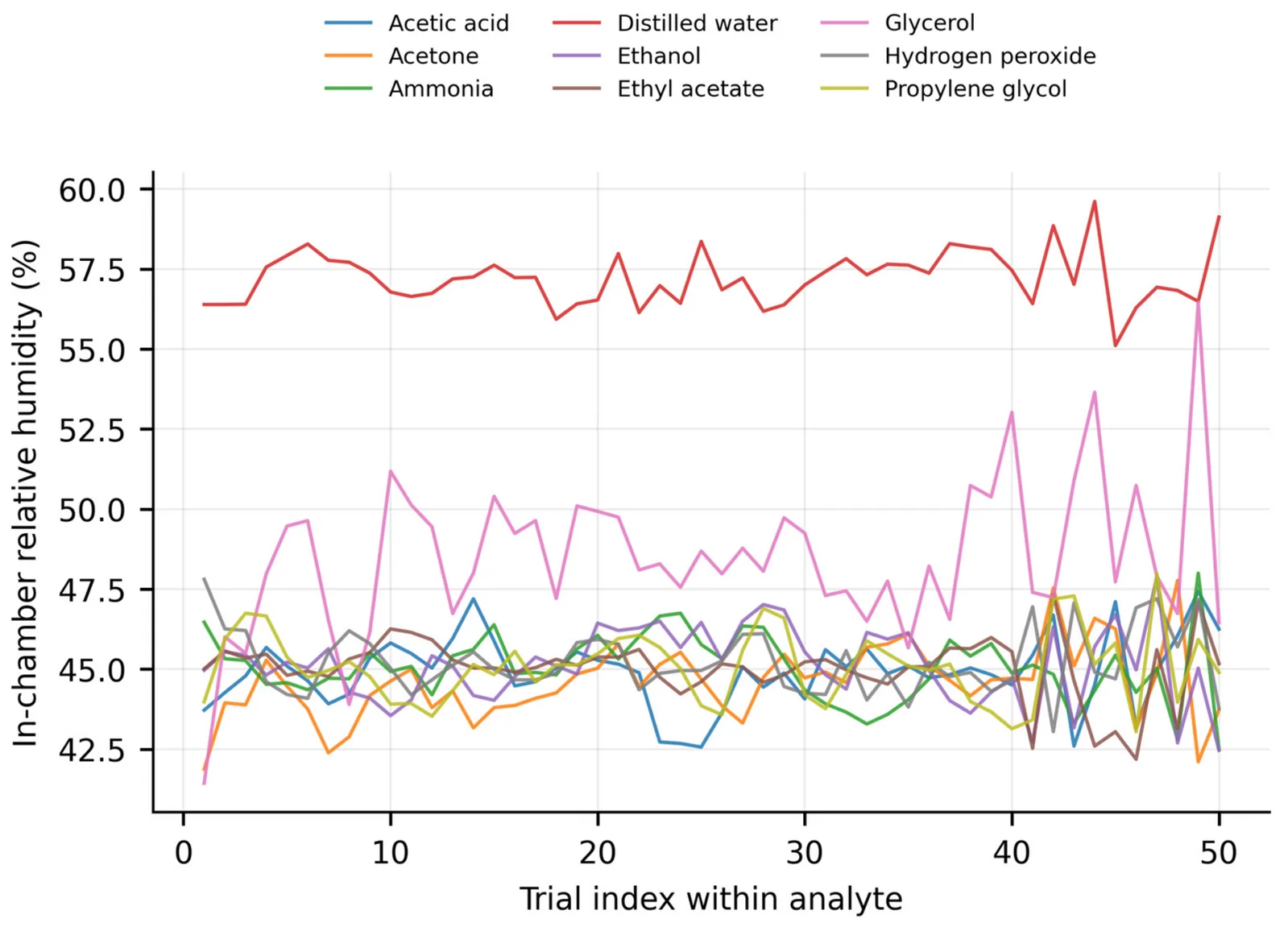
BME680 in-chamber humidity against trial index within analyte on the primary dataset (*n* = 450). The channel shows the extensive between-class overlap expected of a physical measurement and no systematic within-class drift, consistent with the interleaved trial order distributing baseline drift across classes.

Fitted individually across the nine classes, temperature reached 20.4% accuracy and eCO_2_ 39.6%, against 29.1% for humidity. The aggregate figures are less informative than the per-class structure: humidity gives F1 0.980 for distilled water and 0.692 for glycerol while averaging 0.132 across the other seven analytes, whereas temperature and eCO_2_ give 0.216 and 0.141 respectively for water, with no comparable concentration on the confused pair. The gain is therefore attributable to measuring the interferent, not to widening the feature vector. The humidity channel itself shows no systematic trend with trial index within analyte and the between-class overlap expected of a physical measurement (Figure 7), so the gain is not an artifact of acquisition order.

### 3.8. Humidity Distribution by Analyte

**Figure 8:**
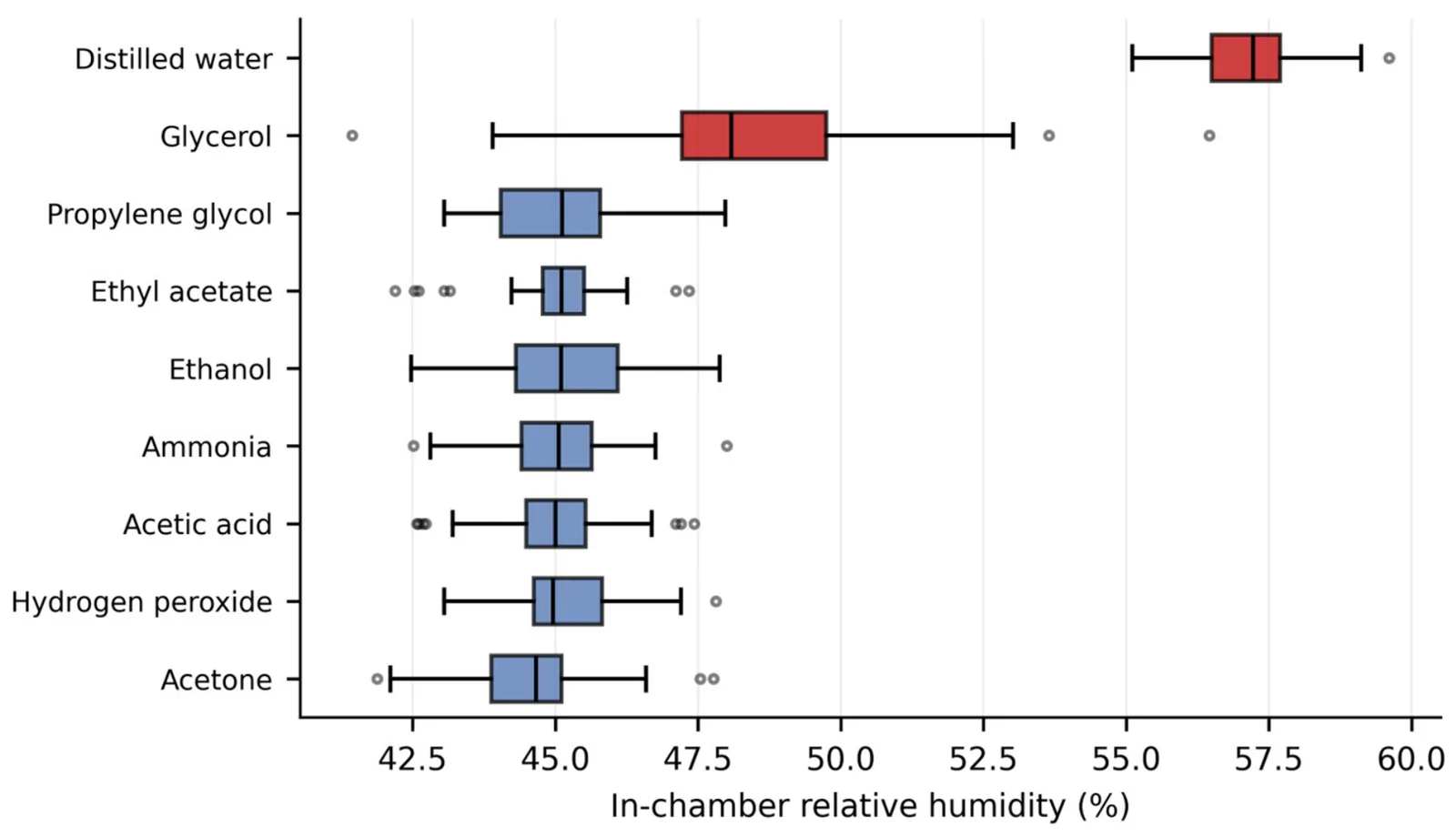
Per-analyte in-chamber relative humidity during acquisition on the primary dataset (*n* = 450). Boxes span the interquartile range (IQR), whiskers extend to 1.5 × IQR, points beyond are plotted individually. Distilled water and glycerol are drawn in red; the seven cleanly classified analytes in blue. Glycerol is elevated relative to those seven and far more variable, and its single highest trial reaches into the range spanned by distilled water.

The per-analyte in-chamber humidity distributions show the pattern that the mechanism predicts (Figure 8). Seven analytes cluster tightly, with medians between 44.7% and 45.1% RH and interquartile ranges under 2 percentage points. The distilled-water control sits well above them at a median of 57.2%, which coincides to one decimal place with the mean quoted in Section 3.6; the two differ by 0.01 percentage points. Glycerol falls between the two groups at a median of 48.1%, but is distinguished less by its median than by its spread: its trials range from 41.4% to 56.5% RH, against a typical range of about 5 points for the seven cleanly classified analytes, and its single highest trial, at 56.5% RH, reaches into the range spanned by the distilled-water control.

Pearson correlations between channels show why the MOS array cannot recover this on its own. In-chamber humidity correlates with MQ-3 at *r* = −0.32 and with MQ-135 at *r* = −0.35, so the array is partially sensitive to water vapor, but that sensitivity is entangled with each channel’s response to the analyte itself. The BME680 measures the same variable without that entanglement, which is why a channel carrying only 12.1% of the impurity-based importance recovers nine percentage points of accuracy.

### 3.9. Sample Size and Learning Behavior

**Figure 9:**
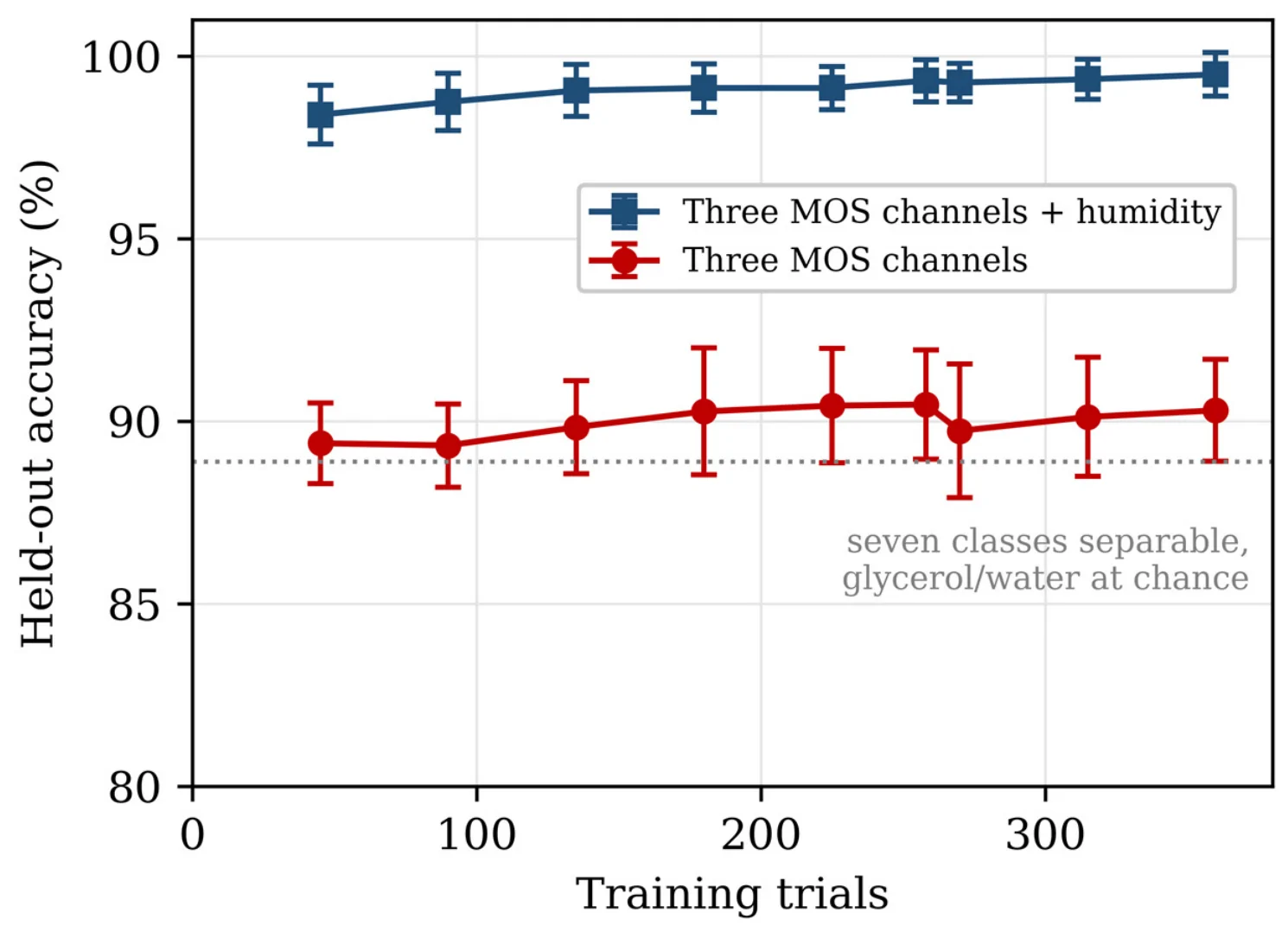
Cross-validated accuracy against training-set size for the three-channel and four-channel feature sets using five-fold stratified cross-validation. Error bars are the standard deviation across folds. Both curves are flat from the smallest training size evaluated; the dotted line marks the accuracy predicted by seven classes separated perfectly and the glycerol–water pair assigned at chance.

Cross-validated accuracy as a function of training-set size (Figure 9) shows that both feature sets reach their asymptotic performance early. The three-channel model is at 89.4 ± 1.1% with 45 training trials and 90.3 ± 1.4% with 360, and the four-channel model is at 98.4 ± 0.8% and 99.5 ± 0.6% over the same interval. Neither curve gains more than about one percentage point beyond the smallest training size evaluated, and the two representations are separated by roughly nine points at every size.

The flat three-channel curve is set by the glycerol–water confusion rather than by data volume. At 258 training trials, per-class held-out accuracy is 98–100% for seven of the nine analytes and 55.8% for glycerol and 61.0% for distilled water; admitting humidity raises the same two classes to 97.2% and 99.2% while leaving the other seven unchanged. Seven classes classified perfectly and two assigned at chance predict 88.9% overall, which is what the three-channel curve shows at every size. The ceiling is imposed by a pair the array cannot separate, and additional trials of that pair did not move it across the range evaluated.

Because both curves are flat across the range evaluated, the nine-point gap between feature representations is a property of the representations on this panel rather than an artifact of dataset size, and the reported accuracies are not limited by the number of trials collected. This holds within the experimental structure evaluated here. Both curves resample trials drawn from the same two preparations per analyte, so they measure sensitivity to trial count and not to the number of independent preparations, which is the quantity a reader should expect to matter more. Per-fold accuracies are given in Table 7; the four-channel model reached 100% in three of five folds and 98.9% in the remaining two.

### 3.10. Stability Across Random Seeds

The four headline feature sets were re-evaluated across ten random seeds, each re-randomizing fold assignment and forest bootstrap with folds aligned within a seed (Table 8). The two results have opposite robustness. Humidity fusion is stable across seeds: its accuracy range is under half a point, and McNemar *p* lies between 5 × 10^−11^ and 7 × 10^−9^ at every seed. Normalization is not: R_s_/R_0_ accuracy spans 5.7 points across seeds, nearly three times the widest spread of any other feature set, and the raw-versus-R_s_/R_0_ comparison reaches significance at two of ten seeds. The representative seed 42 lies within the ten-seed range for every feature set, coinciding with the maximum for three-channel-plus-humidity, which is why the seed-averaged 99.3% rather than the 99.6% of that seed is used as the headline.

## 4. Discussion

### 4.1. Why Normalization Did Not Help, and How Far That Result Reaches

The result follows largely from the structure of the transform. On a single device, the load resistance R_L_ is fixed, so Equation (1) maps each divider voltage to a sensor resistance through a strictly monotone function: R_s_ decreases as V_out_ increases. A tree ensemble partitions each feature by thresholds and is invariant to any strictly monotone transformation of a feature, because such a transformation preserves the ordering on which every split depends. Converting voltage to resistance therefore cannot change what a random forest learns, and the empirical control confirms it: resistance without the R_0_ division matched raw voltage to within 4 of 245 trials (1.6 points) at every seed, the residual reflecting randomized split selection rather than information (Section 3.5). For the forest, the whole effect of resistance-ratio normalization is the per-trial division by R_0_, and that division can help only if between-trial variation in R_0_ is dominated by uninformative drift. The scale-sensitive classifiers make the same point from the other side, since for them the resistance conversion alone slightly improved accuracy while the R_0_ division cost 8–9 points. Whether that division helps depends on what it removes. Normalization suppresses between-trial variation in the clean-air baseline, and that variation has two components: drift, which is uninformative and which normalization is designed to suppress, and any covariance between baseline state and analyte identity, which under these conditions is partly informative. Because trials were interleaved across analytes and acquired over a small number of sessions in an unconditioned laboratory, the baseline drifted relatively little within a session while in-chamber humidity varied, so the drift component that normalization suppresses is small while the division itself injects the measurement noise of R_0_ into every feature. The forest shows the smallest effect because it can discount a noisy feature by splitting on it less, which a linear model cannot do.

Two constraints bound this result. The first is statistical power: with 245 trials, the random-forest comparison alone can detect a large effect but not a small one, and its mean decrease of 3.8 points, significant at the trial level at two of ten seeds, would by itself be consistent both with a modest real cost and with no effect. The linear classifiers resolve that ambiguity: the same division costs them 8–9 points, significant at eight and ten of ten seeds, which is more consistent with a real cost that the forest registers less of than with no cost at all. The second is a confound we cannot exclude with these data. R_0_ was recorded immediately before each exposure, at the end of the 30 s baseline phase that follows the 120 s purge, and for strongly adsorbing analytes the live trace was sometimes still drifting at that point. A baseline that has not fully returned adds noise to every normalized feature and, in the worst case, analyte-dependent bias. Randomized interleaving distributes any carryover across classes rather than confounding it with class identity, so the expected effect is added variance rather than systematic bias, which is consistent with the increased across-seed variability we observe. We did not measure R_0_ recovery directly, however, and cannot rule out that the cost reflects the quality of the baseline estimate rather than the transform itself. A direct test would record the sensor resistance trajectory through an extended purge on a subset of trials and repeat the ablation with R_0_ taken at asymptote. These two constraints are not independent. If the baseline estimate is the source of the cost, then the cost is real but attributable to the protocol rather than to the transform, and our data cannot separate those two readings.

The seed sweep sharpens this picture. At the representative seed, R_s_/R_0_ features showed a smaller fold-to-fold spread than raw voltage (1.5 against 5.3 points), which would suggest normalization reduced between-fold variance at a cost in accuracy. Across seeds, however, R_s_/R_0_ accuracy was the more variable of the two (range 5.7 against 2.0 points), so the single-seed fold spread is not a stable property of the representation. The stable observations are that raw voltage is better on average by about four points, that the R_s_/R_0_ result depends on which trials fall in which fold, and that the dominant error mode is unchanged. All three are consistent with a transform that removes real variation along with drift and injects R_0_ measurement noise whose effect varies with fold composition. This account predicts where normalization should help. Over longer deployments, across physically distinct devices with differing load resistors, or where heater aging accumulates, the drift component grows, and the trade should reverse. Our data test none of those regimes, so the result should be read as bounded: on one device, one panel, and 245 trials, resistance-ratio referencing conferred no benefit and imposed a measurable cost. It does not establish that the technique is unhelpful in general, and it does not contradict the datasheet-level rationale for it.

The ethanol–ethyl acetate confusion amplified under normalization is consistent with the same mechanism. These two analytes are separable under raw voltage by a margin that is small relative to the noise added by per-trial division, so they are the first pair to be lost when that noise is introduced.

### 4.2. What the Nine-Class Accuracy Does and Does Not Measure

A reader could object that the 99.3% figure resolves a confusion the panel design created: glycerol is included alongside a distilled-water control, the glycerol headspace is on our evidence water-dominated, and a humidity sensor separates water from not-water. That objection is correct as a description, and we accept it. Seven of the nine classes are separated by three MOS channels alone, so the panel does not test the array’s discriminative capacity, and the final accuracy should not be quoted as evidence of it.

What the panel does test is attribution. Because the three-channel error is concentrated in one pair, each intervention can be scored on that pair directly: normalization did not move it, temperature and eCO_2_ did not move it, humidity resolved it, and humidity alone carries information about nothing else. That chain of matched comparisons is the finding. The same chain would be uninterpretable on a harder panel with diffuse errors, where any gain from a fourth channel could reflect several mechanisms at once. The glycerol–water pair is also not contrived: glycerol is a common excipient and humectant, several analytes in our own panel are aqueous, and any MOS array sampling aqueous preparations will encounter headspaces whose composition is close to that of water. That only glycerol confused with the control here is consistent with its headspace being close to the control rather than with any special property of glycerol.

### 4.3. Sensor Fusion Against Feature Transformation

The glycerol–water result and the normalization result are two halves of one finding. A feature transform can only reweight information the array already carries. If two headspaces are indistinguishable from three MOS channels because both are water-dominated, no transform of the single scalar those channels supply per trial will separate them. This argument is bounded by the representation: a transform of the response transient, which we did not evaluate, draws on information the scalar discards and is not covered by it. Adding a transducer that measures the distinguishing variable does, and does so almost completely.

We note the interpretive ambiguity that the same humidity reading serves as both the explanation and the remedy, and that the argument would be circular had humidity been introduced as a feature without the independent per-analyte evidence presented first. The order matters: the humidity distributions point to the mechanism, the failure of normalization supports a physical rather than a signal-conditioning origin, and the fusion result is the predicted consequence. The specificity test in Section 3.7 narrows the remaining gap: two other channels of the same kind, admitted the same way, do not produce the gain.

This distinction separates our result from humidity compensation, the established remedy for the same interference. Compensation transforms the MOS signal using a model of the humidity response, whether through a mechanism-based correction [29] or through temperature modulation with discriminant analysis that separates humidity and analyte onto different axes [28]; Cai et al. report an e-nose improving from 87.7% to 99.3% by this route [32]. Our finding is not that compensation fails but that a transform confined to the MOS channels cannot recover what those channels never encoded: resistance-ratio referencing is itself such a transform, and it did not separate the pair. Admitting the interferent as an independent input does. Compensation and fusion are therefore complementary rather than competing, and the choice between them turns on whether the discriminating variable is present in the array’s own response. The rule that follows is narrow, and we offer it as such. On the array and panel tested here, where water vapor is a shared component of most headspaces, adding a humidity transducer was more effective than the resistance-ratio transformation of the MOS channels. We state this as a hypothesis for commodity MOS arrays sampling aqueous preparations rather than as a general result: it rests on one normalization method, one device, one nine-analyte panel and one feature representation, and a transform operating on the response transient rather than on a single scalar per channel was not tested. A BME680 costs a small fraction of the array it supplements.

### 4.4. On-Device Inference

All acquisition, feature extraction, and inference execute on the Raspberry Pi 5 with no network dependency, at under 1 s per trial from end of exposure to class assignment. The comparison datasets in the low-cost electronic nose literature are typically acquired on embedded hardware and then classified off-device [19,20,21,22,23,24,25], though single-board-computer builds are increasingly common [43]. What distinguishes the present system is not the compute platform but the combination of a minimal three-channel array with inference inside the acquisition loop. Removing the network dependency matters for three reasons beyond latency: the instrument remains functional without connectivity, no sample-derived data leaves the device, and the deployed cost does not include a service subscription. The trained forest occupies a few hundred kilobytes and imposes no meaningful power or memory burden relative to the continuously powered sensor heaters, which dominate the platform’s energy budget.

### 4.5. Cost

The USD 83 figure is the total component cost of the build. It is roughly three orders of magnitude below benchtop chromatography and comparable to or below the larger MOS-array systems in the comparison literature, which use five to sixteen sensors. An itemized bill of materials is deposited with the repository. The figure is component cost only, excluding shipping, tax, tools and the 3D printer, and the sensor breakouts are generic parts rather than branded equivalents, which cost roughly three times as much. The dominant single item is the single-board computer; the three MQ sensors, the ADC, and the two digital sensors together account for a minority of the total, which is why adding the humidity transducer is inexpensive relative to the accuracy it recovers.

### 4.6. Limitations

Several constraints bound these results. The headline three-channel accuracy and the raw-versus-normalized comparison come from different acquisitions, so they are not matched trial-for-trial; the ablation is reported on its own dataset, where both representations are available for the same trials, and no cross-dataset comparison is drawn.

The humidity fusion result is obtained on the same trials that motivated it. It awaits confirmation on an independent panel, and we present it as a confirmed prediction rather than as an independently validated performance claim.

Each analyte was tested at a single nominal concentration. The concentration–response behavior of the array is therefore uncharacterized, no limit of detection is reported, and generalization to other concentrations is unverified. Thirty minutes was allowed for headspace equilibration, which for the least volatile analytes, notably glycerol, may not represent full equilibrium, and headspace vapor concentrations were not independently verified for any analyte.

A further route we did not take is temperature modulation, in which the sensor heater is driven through a cycle so that a single element yields a time-resolved response with greater intrinsic selectivity [44]; that approach trades acquisition time for information and would be the natural alternative to adding a transducer. The feature vector comprises a single scalar per channel, discarding the temporal structure of the response transient, which carries additional discriminative information. Return of sensor resistance toward the clean-air baseline, judged from the live trace rather than measured, took up to 120 s or longer for strongly adsorbing analytes such as ammonia, limiting the achievable duty cycle for continuous sampling. The panel comprised pure headspaces, so performance under real-world interferents and matrix effects is untested. The mechanism inferred here predicts which interferents would be most troublesome: any preparation whose headspace is water-dominated, because the liquid is involatile and hygroscopic or because the analyte is dissolved in water, will present to the three MOS channels much as glycerol did. Sorbitol and other involatile polyols, concentrated sugar and salt solutions, aqueous surfactants, and dilute aqueous solutions of any analyte are the obvious cases, and a humidity channel distinguishes them from water only to the extent that their water activities differ. The boundary is not volatility alone: propylene glycol is also a polyol of low volatility and was classified without error here, so the relevant condition is that the headspace carries too little analyte vapor for the MOS channels to register, not that the liquid be involatile. Conversely, analytes that are themselves volatile and chemically close to a panel member, for instance methanol or isopropanol against ethanol, would be expected to confuse the array for the opposite reason, and no auxiliary transducer on this deck would resolve them; arrays of comparable size have been characterized on defined mixtures of volatile organic analytes [31].

Trials within an acquisition batch derive from a shared sample preparation, so the preparation rather than the trial is the independent replication unit. Because the two batches are also the two preparations, the cross-day holdouts train and test on separate preparations and are therefore preparation-level estimates: 86.7% for three channels and 93.3% with humidity on the primary dataset, and 85.4% against 77.2% for raw and normalized features on the ablation. These are the figures we regard as the primary performance estimates. The shuffled cross-validation results should be read as upper bounds. With only two preparations per analyte, the preparation-level estimates are themselves based on a single train–test split and carry no confidence interval over preparations; more preparations are needed before a preparation-level variance can be reported. All results were obtained on a single fabricated device; device-to-device variation in MQ sensors is substantial and is untested here. Finally, chemical specificity remains far below that of chromatographic methods: the array resolves chemical classes, not molecular identity, and cannot substitute for GC–MS where molecular assignment is required.

The platform also carries a CCS811 TVOC channel that was withheld from every feature set and every reported result. Fitted alone, it classified all nine analytes at 100.0%, against 76.0% for MQ-3 at the representative seed, the best physical channel (77.0 ± 1.4% across ten seeds, Section 3.3), and its per-class distributions separate more sharply than any physical channel on the deck. We could not account for that behavior, although three checks constrain it. The first is that the separation follows from a class-mean ratio of 8.7, against 1.9 for MQ-3, rather than from greater measurement precision, since the median within-class coefficient of variation for TVOC is 1.33%, inside the 1.22–2.44% range spanned by every other channel. The second is that the three volatile organic solvents occupy the top three positions and the two water-dominated headspaces the bottom two, the grouping a VOC-responsive channel should produce and one consistent with the water-dominated glycerol headspace inferred in Section 3.6, although the ordering does not track vapor pressure strictly (Table S4). The third is that the channel is not a rescaling of the eCO2 output of the same device: the two share trial-to-trial variation within analyte at a median of *r* = +0.52, but the correlation between their class means is *r* = +0.001, and a monotone rescaling would have preserved class ordering. The most likely mechanism is a proprietary firmware transform whose output range is wide relative to its noise, but the transform is undocumented, and the device reports TVOC relative to an internal clean-air baseline that was not logged during acquisition, so we cannot confirm it. Because a channel we cannot account for raises the question of whether the same effect touches the reported channels, we quantified per-class separation for every channel by one-way *F* statistic and by the smallest inter-class gap across all 36 class pairs; the signature is confined to TVOC, and the three reported MOS channels and the humidity channel show none of it. The full account, with the supporting tables, is given in the Supplementary Materials (Section S1, Tables S1–S4). We retain the TVOC channel as unverified rather than exploit or interpret it; the raw values are deposited with the repository so that others can examine them.

## 5. Conclusions

On a three-channel commodity MOS array with fully on-device inference, we isolated the contributions of two design choices that the low-cost electronic nose literature often applies but does not measure. On a single device, converting divider voltage to sensor resistance is a monotone transform that a random forest cannot see, so resistance-ratio referencing reduces to a division by the per-trial clean-air baseline. That division conferred no benefit and imposed a consistent cost: 3.8 points for a random forest, seed-sensitive at the trial level, and 8–9 points for two linear classifiers, significant at nearly every seed; incomplete baseline recovery may contribute to that cost and cannot be excluded with these data. Independent humidity measurements support a physical rather than a signal-conditioning origin for the dominant residual error mode, a glycerol–water confusion, and admitting a humidity channel as a fourth feature resolves it as predicted while two other auxiliary channels do not. Under shuffled cross-validation, nine-class accuracy rises from 90.2 ± 0.5% to 99.3 ± 0.2% (mean ± s.d. across ten seeds), a gain that is significant at every seed; under the conservative preparation-level holdout, it rises from 86.7% to 93.3%.

Seven of the nine headspaces are resolved by three sensors alone, and we do not present the final accuracy as evidence of instrument capability. A leave-one-sensor-out test placed MQ-9 at 0.89 ± 0.63 points on three channels and at no measurable contribution once humidity is admitted, so on this panel the minimal configuration this result points to carries MQ-3, MQ-135 and a humidity transducer rather than three MOS channels. The contribution is the matched attribution of each gain, and the design rule we propose from it: where an array’s residual error is traced to a variable the array does not measure, add a transducer for that variable rather than transform channels that do not encode it. We tested this on one device, one panel of nine pure headspaces and one normalization method, and it should be read as a hypothesis for commodity MOS arrays sampling aqueous preparations rather than as an established rule. The platform costs USD 83 in components, and the seven classifiers evaluated on this dataset performed comparably, within about 2 points of one another. Its accuracy falls on a held-out day and preparation, from 99.3% under cross-validation to 93.3% for the four-channel model and from 90.2% to 86.7% for three channels, so the cross-validated figures overstate performance on new preparations.

The immediate next steps are confirmation of the fusion result on an independent analyte panel, direct measurement of baseline recovery during the purge to close the confound on the normalization result, heated headspace generation with independently verified vapor concentration to test the physical account of the glycerol–water confusion directly, concentration series to establish detection limits, replication across fabricated devices, and features derived from the response transient to test how much discriminative information the current single-scalar representation discards. Two of these bear on whether the array would work outside this panel, and we state them as requirements rather than as options. The first is homologous series: every headspace tested here was a pure substance, and the panel members are chemically distinct, so an array that separates ethanol from ethyl acetate has not been shown to separate ethanol from methanol or propanol, which present the MOS channels with closely related reducing species. Section 4.6 names this as the error mode that no auxiliary transducer on this deck would resolve, and it must be tested directly rather than assumed. The second is concentration: each analyte was presented undiluted at a single nominal concentration, which is not the regime of a real sample, so classification must be verified across the concentration range that the intended application actually presents. Until both are done, the results reported here characterize the instrument on a panel of pure substances and nothing beyond it. Evaluation on real-world samples containing interferents is required before any application claim can be made.

## Figures and Tables

**Table 1:** Representative low-cost MOS electronic nose systems. *n* is the number of classes; “Isolated” indicates whether the signal-conditioning step and the auxiliary channel were each ablated against a matched baseline on the same trials. T/RH, temperature and relative humidity transducers.

System	Sensors	*n*	Inference	Isolated
Wijaya [19]	11 MOS	4	Off-device	No
Sarno [20]	8 MQ, T/RH	7	Off-device	No
Fonollosa [23]	16 MOS	6	Off-device	No
War [26]	5 MQ, T/RH	2	Off-device	No
SCENT	3 MQ, RH	9	On-device	Yes

**Table 2:** Per-class performance with the three MOS channels on the primary dataset (*n* = 450) using pooled out-of-fold predictions at seed 42.

Analyte	*n*	Precision	Recall
Acetic acid	50	1.000	1.000
Acetone	50	1.000	0.980
Ammonia	50	1.000	1.000
Distilled water	50	0.551	0.540
Ethanol	50	0.980	1.000
Ethyl acetate	50	1.000	1.000
Glycerol	50	0.549	0.560
Hydrogen peroxide	50	1.000	1.000
Propylene glycol	50	1.000	1.000

**Table 3:** Comparison of classifier families on the same three features (*n* = 450) at seed 42; ± is fold-to-fold s.d. RBF, radial basis function kernel.

Classifier	Accuracy (%)	Macro F1
Support vector machine (RBF)	91.8 ± 2.6	0.917
Support vector machine (linear)	91.8 ± 2.6	0.917
Gaussian naive Bayes	91.3 ± 2.6	0.911
Logistic regression	90.9 ± 1.8	0.908
k-nearest neighbors (k = 5)	90.7 ± 2.1	0.904
Decision tree	90.4 ± 1.1	0.903
Random forest (100 trees, deployed)	89.8 ± 0.8	0.897

**Table 4:** Effect of resistance-ratio normalization on the ablation dataset (*n* = 245). Accuracy is given as mean ± s.d. across ten seeds with range in parentheses; F1 and recall columns are from pooled out-of-fold predictions at seed 42. “Other 7” is the mean recall across the seven analytes other than glycerol and distilled water.

Features	Acc. (%), 10 Seeds	Macro F1	Gly F1	Wat F1	Other 7
Raw divider voltage	86.2 ± 0.9 (85.3–87.3)	0.882	0.592	0.551	0.971
R_s_/R_0_ normalized	82.4 ± 1.9 (79.2–84.9)	0.823	0.535	0.486	0.914

**Table 5:** Per-class performance with humidity as a fourth feature (*n* = 450) using pooled out-of-fold predictions at seed 42.

Analyte	*n*	Precision	Recall
Acetic acid	50	1.000	1.000
Acetone	50	1.000	0.980
Ammonia	50	1.000	1.000
Distilled water	50	0.980	1.000
Ethanol	50	0.980	1.000
Ethyl acetate	50	1.000	1.000
Glycerol	50	1.000	0.980
Hydrogen peroxide	50	1.000	1.000
Propylene glycol	50	1.000	1.000

**Table 6:** Effect of each auxiliary channel on the primary dataset (*n* = 450) at seed 42; ± is fold-to-fold s.d. Seed-averaged values for the first and last rows are given in Section 3.10.

Feature Set	Acc. (%)	Gly F1	Wat F1	*p* vs. 3 MOS
Three MOS channels	89.8 ± 0.8	0.554	0.545	—
+temperature	90.9 ± 2.3	0.592	0.608	0.33
+eCO_2_	90.9 ± 2.7	0.615	0.583	0.36
+humidity	99.6 ± 0.5	0.990	0.990	<0.001

**Table 7:** Per-fold cross-validated accuracy (%) on the primary dataset (*n* = 450) at seed 42.

Fold	Three MOS Channels	+BME680 Humidity
1	90.0	100.0
2	90.0	98.9
3	91.1	100.0
4	88.9	98.9
5	88.9	100.0
Mean ± s.d.	89.8 ± 0.8	99.6 ± 0.5

**Table 8:** Headline comparisons across ten random seeds (0–9). Accuracy is given as mean ± s.d. with min–max; seed 42 is the representative seed used for the single-seed tables.

Comparison	Seed 42	Mean ± s.d. (10 Seeds)	Min–Max
Three MOS channels, primary (%)	89.8	90.2 ± 0.5	89.3–90.9
+ humidity, primary (%)	99.6	99.3 ± 0.2	99.1–99.6
McNemar *p*, humidity vs. three MOS	<0.001	—	5 × 10^−11^–7 × 10^−9^
Raw voltage, ablation (%)	85.7	86.2 ± 0.9	85.3–87.3
R_s_/R_0_, ablation (%)	80.0	82.4 ± 1.9	79.2–84.9
McNemar *p*, raw vs. R_s_/R_0_	0.076	median 0.24; *p* < 0.05 at 2 of 10	0.03–1.00

## Data Availability

The raw sensor logs for both datasets, the acquisition script, the analysis script for the single-seed results, the scripts behind the seed sweep, the leave-one-sensor-out ablation, the learning curve and the CCS811 exclusion checks together with their outputs, the chamber STL and Fritzing wiring sketch (Fritzing 1.0.6; https://fritzing.org, accessed on 15 October 2026), and the itemized bill of materials are all openly available at https://github.com/Fire-Dev2/SCENT (accessed on 15 October 2026) and archived at https://doi.org/10.5281/zenodo.22244532 (accessed on 15 October 2026), with nothing withheld from either deposit.

## Supplementary Materials

The following supporting information can be downloaded at: https://www.mdpi.com/article/10.3390/s26206645/s1. Table S1: Per-channel class separation on the primary dataset (*n* = 450); Table S2: Correlation of each reported channel with the CCS811 TVOC channel, primary dataset, pooled across all 450 trials; Table S3: Spearman correlation of each channel with trial index within analyte, primary dataset; Table S4: CCS811 TVOC class means in descending order, primary dataset; Table S5: Leave-one-sensor-out ablation, primary dataset (*n* = 450), ten random seeds; Table S5b: Per-class F1 under each dropped channel, three-channel model at the representative seed; Table S5c: Leave-one-sensor-out ablation under the preparation-level holdout, primary dataset; Section S1: Extended account of the CCS811 TVOC channel.
