# Supplementary material for: Isolating the Contributions of Resistance-Ratio Normalization and Humidity Fusion in a Minimal Three-Sensor Electronic Nose
Source: Sensors (Basel). 2026 Oct 21;26(20):6645. doi: 10.3390/s26206645 (PMC13709548; doi:10.3390/s26206645)
Supplement: Supplementary file 1 [file sensors-26-06645-s001.zip › sensors-4609768-supplementary.pdf]

sensors

MDPI

Supplementary Materials

Isolating the Contributions of Resistance-Ratio Normalization and Humidity Fusion in a Minimal Three-Sensor Electronic Nose

Tharun H. Nambi, Nikhil Bhimireddy and Joseph P. McElroy

All values are computed by the scripts deposited at https://github.com/Fire-Dev2/SCENT. Tables S1 to S4 are produced by analysis/tvoc_checks.py and Tables S5, S5b and S5c by analysis/loso.py. This file has no reference list of its own; bracketed numbers refer to the manuscript reference list and the sources are named in full where they appear.

Table S1. Per-channel class separation on the primary dataset (n = 450). F is the one-way statistic across the nine analytes. The minimum pair gap is the smallest separation between any of the 36 class pairs, in pooled standard deviations; a negative value means the two classes overlap. The coefficient of variation is the median within-class value across the nine analytes, and the class-mean ratio is the largest class mean divided by the smallest.

Channel	F	Min pair gap (s.d.)	Overlapping pairs	Median within-class CV (%)	Class-mean ratio
MQ-3	16,209.2	−1.812	3/36	1.24	1.91
MQ-9	3,393.6	−1.898	13/36	1.52	1.41
MQ-135	10,489.2	−1.968	4/36	1.32	1.96
BME680 humidity	502.9	−1.982	25/36	2.44	1.28
BME680 temperature	54.0	−1.962	27/36	2.15	1.05
CCS811 eCO2	855.0	−1.967	11/36	1.22	1.17
CCS811 TVOC	87,945.1	+7.692	0/36	1.33	8.72

CCS811 TVOC is the only channel with no overlapping class pair, and its F statistic exceeds the next-highest channel by a factor of 5.4. Its within-class dispersion is ordinary; the separation comes from a class-mean ratio of 8.72, against 1.91 for MQ-3.

Table S2. Correlation of each reported channel with the CCS811 TVOC channel, primary dataset, pooled across all 450 trials. The decomposition of the TVOC-against-eCO2 correlation between and within classes is given in the note below the table.

Channel	Pearson r	Spearman rho
MQ-3	+0.959	+0.777
MQ-9	−0.065	+0.258
MQ-135	−0.175	+0.226
BME680 humidity	−0.464	−0.513

The CCS811 TVOC and eCO2 outputs are derived by firmware from a common sensor resistance and share trial-to-trial variation within analyte, with a median within-analyte correlation of r = +0.517. Their class-level structure is unrelated: the correlation between class means is r = +0.001, and pooled over all trials the two components cancel to r = +0.003. A monotone rescaling would preserve class ordering, so eCO2 is not a rescaling of TVOC.

Table S3. Spearman correlation of each channel with trial index within analyte, primary dataset. A systematic acquisition-order artifact would appear here as a consistent non-zero median.

Channel	Median rho	Max |rho|	Analytes with $p < 0.05$
MQ-3	+0.068	0.466	1/9
MQ-9	−0.117	0.391	2/9
MQ-135	+0.004	0.519	2/9
BME680 humidity	−0.000	0.391	1/9
CCS811 TVOC	+0.119	0.612	1/9

No channel shows a consistent trend with trial index, CCS811 TVOC included, so acquisition order does not account for its separation.

Table S4. CCS811 TVOC class means in descending order, primary dataset. The three volatile organic solvents occupy the top three positions and the two water-dominated headspaces the bottom two, the grouping a VOC-responsive channel should produce. The ordering does not track vapor pressure strictly: propylene glycol ranks above both acetic acid and ammonia despite a far lower vapor pressure, and acetone, the most volatile of the solvents, ranks third.

Analyte	Mean TVOC (ppb)
Ethyl acetate	2,178.7
Ethanol	1,949.4
Acetone	1,529.6
Propylene glycol	931.7
Acetic acid	678.5
Ammonia	609.4
Hydrogen peroxide	454.8
Glycerol	334.5
Distilled water	250.0

Recorded values span 242 to 2294 ppb across both datasets, well within the output range specified in the datasheet revision cited in the manuscript.

Table S5. Leave-one-sensor-out ablation, primary dataset (n = 450), ten random seeds. Accuracy is mean and standard deviation across seeds, with the observed range. Cost is the drop from the corresponding full model. Impurity importance credits every channel, so necessity is tested by refitting without each one.

Channels retained	Accuracy (%)	Range (%)	Cost (points)
All three MOS channels	90.18 ± 0.49	89.33–90.89	reference
Without MQ-3	81.60 ± 0.68	80.44–82.44	+8.58
Without MQ-9	89.29 ± 0.83	87.78–90.89	+0.89
Without MQ-135	87.56 ± 0.75	86.00–88.44	+2.62
MQ-3 alone	77.00 ± 1.39	74.44–78.67	
MQ-9 alone	35.16 ± 0.90	34.00–36.44	
MQ-135 alone	65.42 ± 1.09	63.56–66.89	
All three MOS plus humidity	99.33 ± 0.18	99.11–99.56	reference
Without MQ-3, with humidity	91.98 ± 0.67	90.67–92.67	+7.36
Without MQ-9, with humidity	99.47 ± 0.16	99.33–99.78	−0.13
Without MQ-135, with humidity	98.16 ± 0.35	97.56–98.89	+1.18
Three MOS channels, without humidity	90.18 ± 0.49	89.33–90.89	+9.16

Taken as paired per-seed differences, dropping MQ-9 costs 0.89 ± 0.63 points on three channels, with the two-channel model the more accurate at one of ten seeds, and −0.13 ± 0.16 points on four, a difference never positive at any seed. MQ-9 is therefore close to redundant on three channels and makes no measurable contribution once humidity is admitted, on this panel. Dropping MQ-3 costs 8.58 ± 0.69 points and dropping MQ-135 2.62 ± 0.63. The cost column above is the difference of the seed means; the paired differences quoted here are computed seed by seed and carry the correct uncertainty.

Table S5b. Per-class F1 under each dropped channel, three-channel model at the representative seed.

**Analyte**	**All three**	**Without MQ-3**	**Without MQ-9**	**Without MQ-135**
Acetic acid	1.000	1.000	1.000	0.990
Acetone	0.990	0.596	0.990	0.980
Ammonia	1.000	1.000	1.000	1.000
Distilled water	0.545	0.594	0.549	0.535
Ethanol	0.990	0.990	0.990	0.949
Ethyl acetate	1.000	1.000	1.000	0.970
Glycerol	0.554	0.586	0.531	0.531
Hydrogen peroxide	1.000	1.000	1.000	1.000
Propylene glycol	1.000	0.648	1.000	1.000

Dropping MQ-3 collapses acetone and propylene glycol. Dropping MQ-9 leaves every class within 0.03 of its three-channel value.

Table S5c. Leave-one-sensor-out ablation under the preparation-level holdout, primary dataset. Trained on the first acquisition batch (360 trials) and tested on the held-out second batch (90 trials), prepared and acquired on a different day. Ten forest seeds; the paired cost is the full model minus the reduced model, taken seed by seed.

**Feature set**	**Channels retained**	**Accuracy (%)**	**Paired cost**	**Reduced ≥ full**
Three MOS	all (reference)	87.00 ± 1.96	—	—
	without MQ-3	76.11 ± 1.20	+10.89 ± 2.39	0/10
	without MQ-9	87.89 ± 1.52	−0.89 ± 2.61	8/10
	without MQ-135	84.11 ± 0.54	+2.89 ± 2.11	1/10
Four features	all (reference)	94.33 ± 1.69	—	—
	without MQ-3	86.11 ± 1.08	+8.22 ± 2.17	0/10
	without MQ-9	95.56 ± 0.91	−1.22 ± 1.92	8/10
	without MQ-135	92.56 ± 0.91	+1.78 ± 2.23	4/10

The direction agrees with the cross-validated ablation in Table S5: MQ-3 is essential under both, and removing MQ-9 is not a cost, with the reduced model at least as accurate at eight of ten seeds for both feature sets. The held-out preparation comprises 90 trials, so the intervals are wide and this test constrains the direction of the MQ-9 result rather than its magnitude. Produced by analysis/loso.py.

Section S1. Extended account of the CCS811 TVOC channel

This section carries the full account of the CCS811 TVOC channel. Section 4.6 of the manuscript states the finding and the conclusion; what follows is the evidence behind each step. The content is as submitted except where the manuscript was corrected during revision.

The platform also carries a CCS811 TVOC channel that was withheld from every feature set and every reported result. Its per-class distributions separate far more sharply than any physical channel on the deck: fitted alone it classified all nine analytes at 100.0%,

against 76.0% for MQ-3 at the representative seed, the best physical channel. Three observations bear on the cause. First, the separation reflects the spread of the reported quantity rather than measurement precision. The median within-class coefficient of variation for TVOC is 1.33%, inside the 1.22–2.44% range spanned by every other channel, while its class means span a factor of 8.7 against 1.9 for MQ-3. That difference in spread is expected from what each channel reports: the MQ channels report a divider voltage that compresses concentration through the power-law response of these sensors (Yamazoe and Shimanoe, Sens. Actuators B Chem. 2008, 128, 566–573; reference [17] of the manuscript), whereas the CCS811 reports a concentration estimate in ppb, which is not compressed in the same way.

The larger *F* statistic therefore follows from between-class spread rather than from greater measurement precision, although rescaling every channel to unit coefficient of variation does not by itself remove the difference, and we do not claim that dynamic range accounts for it in full. Second, the class ordering groups as a VOC-responsive channel should: ethyl acetate, ethanol and acetone rank highest, distilled water lowest and glycerol immediately above it, which is consistent with the water-dominated glycerol headspace inferred in Section 3.6. The ordering does not track vapor pressure strictly, however: propylene glycol ranks above both acetic acid and ammonia despite a far lower vapor pressure, and acetone, the most volatile of the three solvents, ranks third among them. Third, the channel is not a rescaling of the eCO2 output of the same device. The two outputs are derived by firmware from a common sensor resistance and do share trial-to-trial variation within analyte, correlating at a median of $r = +0.52$, but their class-level structure is unrelated: the correlation between their class means is $r = +0.001$, and pooled over all trials the two components cancel to $r = +0.003$.

A monotone rescaling would preserve class ordering, and the eCO2 channel does not reproduce the ordering of TVOC. The most likely mechanism is therefore that the CCS811 firmware maps sensor resistance to a TVOC estimate through a transform whose output range is wide relative to its noise, expanding between-class differences while leaving relative within-class dispersion unchanged. The recorded values, 242–2294 ppb across both datasets, lie well within the 0–32,768 ppb output range specified in the revision of the datasheet cited here (ams AG, CCS811 datasheet v1-06, 2019; reference [42] of the manuscript); earlier revisions specified a narrower range, and values outside it are not evidence of a fault. We cannot confirm the mechanism: the transform is proprietary, and the device reports TVOC relative to an internal clean-air baseline that was not logged during acquisition (same datasheet), so neither the absolute calibration nor any baseline drift can be reconstructed from our records.

Because a channel we cannot fully account for raises the question of whether the same effect touches the reported channels, we quantified per-class separation for every channel by one-way *F* statistic and by the smallest inter-class gap across all 36 class pairs; the signature is confined to TVOC, and the three reported MOS channels and the humidity channel show none of it (Tables S1 to S4 above). We therefore retain the TVOC channel as unverified rather than exploit or interpret it; the raw values are deposited with the repository so that others can examine them.
